# Exploring the association of addiction-related genetic factors with non-suicidal self-injury in adolescents

**DOI:** 10.3389/fpsyt.2023.1126615

**Published:** 2023-03-31

**Authors:** Zhichao Guo, Yilin Liu, Chengjuan Wang, Shujun Li, Lei Yu, Wenzhi Wu, Xu You, Yunqiao Zhang, Zhaowei Teng, Yong Zeng

**Affiliations:** ^1^The Second Affiliated Hospital of Kunming Medical University, Kunming, China; ^2^The Third Affiliated Hospital of Kunming Medical University, Kunming, China; ^3^Honghe Second People's Hospital, Honghe, China

**Keywords:** non-suicidal self-injury, addiction, genes, bioinformatics, biomarker

## Abstract

**Background:**

Non-suicidal self-injury (NSSI) is self-injurious behavior without suicidal intent commonly seen in the adolescent population and poses a serious threat to the life safety of adolescents. Related researches suggest a possible correlation between addiction and the occurrence of NSSI. This study aimed to explore the correlation between addiction and NSSI from a molecular biological perspective by analyzing the differential expression of addiction-related genes in NSSI patients.

**Methods:**

(1) The association between addiction and non-suicidal self-injury in a Chinese adolescent population was verified with the help of questionnaires on substance and non-substance addictions and non-suicidal self-injury among 1,329 adolescents in China, (2) Screening for key genes associated with addiction by bioinformatics analysis, and (3) RT-qPCR experiment was performed to validate key genes and Receiver Operating Characteristic curves were plotted for target genes.

**Results:**

(1) Substance and non-substance addictions were all significantly correlated with non-suicidal self-injury, (2) Four target genes: *SERPINA3*, *SLC14A1*, *RPS6* and *RPS3A* were screened by bioinformatics technique, and (3) Relative quantitative analysis by RT-qPCR revealed that the expression levels of *SLC14A1* (*p* < 0.01), *RPS6* (*p* < 0.05) and *RPS3A* (*p* < 0.01) were significantly higher in NSSI patients than in healthy controls.

**Conclusion:**

(1) The significant association between addiction and NSSI exists in the Chinese adolescent population and (2) Addiction-related genes *SLC14A1*, *RPS6*, and *RPS3A* are differentially expressed in adolescents with NSSI. The genes have the potential to become biological markers for the diagnosis of NSSI.

## 1. Introduction

Non-Suicidal Self-Injury (NSSI) is self-injurious behavior that does not culminate in suicide and uses various methods to cause varying degrees of damage to body tissue, usually cutting, scratching, hitting the body and burning (1).

Although NSSI is clinically considered to be behavior without suicidal intent, it is associated with a high risk of potentially suicidal behavior (2). A study by Liang et al. in mainland China suggests that a high frequency of NSSI events may lead to a significant increase in suicide risk (3). Anestis also found that 33.6% of people with NSSI had attempted suicide, compared to just 2.5% of those without a history of NSSI who would experience suicidal ideation and behavior (4). In addition, some people involved in NSSI may have an increased risk of infection due to uncontrolled self-injury or lack of timely treatment, leading to an increased risk of potential death (5). Consequently, NSSI poses a significant threat to the life safety of individuals and affects health to varying degrees. This has forced NSSI to become a major public health issue that needs to be faced globally. Data from research studies over the last decade show that self-harming behavior is increasing among different populations in various countries around the world, especially among adolescents and young adults (6, 7). According to the statistical results of related studies, the lifetime prevalence of NSSI in children and adolescents is 17–18% worldwide, and only 4–6% in adults (8–10), while the prevalence of NSSI in the Chinese population aged 13–18 years is about 27.4%, followed by those aged 18–22 years (13.6%), far exceeding the world average (11, 12). NSSI is showing a trend toward a younger age group and adolescents are the high prevalence of NSSI and the key target population for NSSI research.

In recent years, the hypothesis of NSSI addiction has come to the attention of more researchers and has been shown to have a strong correlation in more studies (13–15). Substance addiction can play a very critical predisposing role as an important moderator in studying the moderating effects of addictive behaviors on self-harming behaviors (16). Among other things, alcohol as a de-inhibitor of weakened neurological function can trigger impulsivity and loss of control. Substance addiction resulting from alcohol abuse may lead to aggressive behaviors such as self-injury and suicide (17–19). Surprisingly, recent researches in the literature suggest similarities between non-suicidal self-injury and other maladaptive and compulsive behaviors such as non-substance addiction (which shares many features and common neurobiological and genetic underpinnings with substance addiction) (20–22). Among these, smartphone addiction, a hot topic in the field of non-substance addiction, was shown to be significantly correlated with non-suicidal self-injury in a cross-sectional study by Elisa et al. (23). In addition, researchers in clinical samples have reported that individuals with NSSI experience an immediate sense of relief during self-harm similar to that characteristic of addiction, which may be accompanied by a sense of urgency and craving (24). Similarly, significant impairment of executive control functions (25–27) and lower levels of endogenous opioids (e.g., β-endorphins, methionine enkephalins) in the cerebrospinal fluid that regulate pain and addictive behavior has been observed in individuals with NSSI compared to healthy individuals under laboratory condition (28, 29). Despite the results of clinical reports and laboratory studies suggesting a high correlation between NSSI and addiction, there is still a lack of a clear marker from biology to demonstrate the physiological mechanisms underlying the correlation between addiction and NSSI. In addition, gene chips and high-throughput sequencing technology have become one of the hot spots for studying the occurrence, development and prognosis of diseases. In this study, bioinformatics technique combined with molecular biology experiment will be used to mine genetic data from public data platforms to screen out differentially expressed genes associated with addiction. After GO functional annotation and KEGG pathway enrichment analysis, we will verify which genes are significantly differentially expressed by RT-qPCR in peripheral venous blood samples from NSSI patients and healthy individuals. We expect to identify addiction-related molecules that are differentially expressed in NSSI patients and provide useful information for exploring the pathogenesis and therapeutic targets of NSSI.

Most of the past research on addiction and non-suicidal self-injury has been based on populations from Western countries outside of China. Because of ethnic, geographic, and cultural variability, we were unable to determine from data from other countries that addiction and non-suicidal self-injury are also significantly correlated in Chinese populations or even among adolescents. Based on such speculation, in this study, we will select a subset of adolescents in a particular region of China and administer a questionnaire on substance addiction, non-substance addiction and non-suicidal self-injury. We used statistical analyses to determine whether addiction is statistically associated with non-suicidal self-injury in the Chinese adolescent population to provide a theoretical basis for the experimental study.

The current study plans to use a questionnaire to analyze the statistical association between substance/non-substance addiction and non-suicidal self-injury to verify whether addiction is theoretically associated with non-suicidal self-injury in the Chinese adolescent population. Subsequently, bioinformatics techniques combined with molecular biology experiments will be applied to predict addiction-related molecules and validate them in clinical samples to determine their association with non-suicidal self-injury and their potential use as diagnostic markers, thus providing new ideas to investigate the addiction mechanisms and treatment options for non-suicidal self-injury. Based on the findings of previous research and the purpose of this study, we propose the following hypotheses: (1) Addiction and non-suicidal self-injury are significantly associated in the Chinese adolescent population. (2) There is a significant difference in the expression of addiction-related molecules in patients with non-suicidal self-injury and these molecules have the potential to be used as markers for the diagnosis of non-suicidal self-injury.

## 2. Materials and methods

### 2.1. Participants

#### 2.1.1. Questionnaire survey

Two high schools in a city in Yunnan Province, China, were randomly selected for this study, and all students in the senior class of each high school were included in the study (sophomores and juniors were excluded due to high school academic level tests and college entrance exams). After excluding incomplete questionnaires and gender-missing questionnaires, 1,329 valid questionnaires were obtained with an effective rate of 93.4%. Participants ranged from 16 to 18 years old, including 521 boys and 808 girls (M age = 16.5 ± 0.6 years).

Psychology professionals who had undergone rigorous pre-training organized the students to complete the online questionnaire in class *via* the internet, and students were asked to complete the questionnaire completely and independently. The participants were informed in advance that the data collection was anonymous and that the information on the questionnaire was completely confidential. Consent was obtained from schools, parents, and participants for this study. Participants have carefully read and signed the informed consent before participating in this study.

#### 2.1.2. Experiment study

Participants in the experimental group were from the inpatient psychiatric ward of a large tertiary care hospital in Yunnan Province, China. The following were the inclusion criteria for the experimental group participants: (1) Clinical presentation met the diagnostic criteria for non-suicidal self-injury in the DMS-5, (2) Absence of other psychiatric disorders (depression, anxiety, and schizophrenia, etc.), (3) A total score of >0 on the Non-Suicidal Self-Injury Scale, and (4) The presence of non-suicidal self-injury is confirmed in the patient’s medical record written by a licensed clinical psychiatrist.

Participants in the healthy control group were from a healthy population randomly recruited from a region in Yunnan Province, China. The following were the inclusion criteria for the control group participants: (1) Behavioral manifestations did not meet any of the diagnostic criteria for non-suicidal self-injury in the DMS-5, (2) Be in good health and have a normal routine physical examination, (3) No family history of psychiatric disorders, (4) No history of blood product transfusion within 3 months, and (5) Exclude women who are breastfeeding or pregnant.

Informed consent was obtained from all subjects before any relevant study. In cases where a parent or guardian was delegated to decide on the subject’s participation, the parent or guardian signed informed consent on behalf of the subject.

A total of 24 patients with a mean age of 16.0 ± 2.5 years (3 males and 21 females) were recruited for the project; 24 healthy individuals with a mean age of 31.6 ± 11.3 years (12 males and 12 females) were recruited as controls (Supplementary Table S1). The study was reviewed and approved by the Medical Ethics Committee of Kunming Medical University.

### 2.2. Measures

#### 2.2.1. Substance addiction questionnaire

Because the non-clinical sample was drawn from normal, healthy high school students rather than dependent patients and the pre-test results indicated that very few individuals met the criteria for substance dependence, we did not use a professional alcohol/tobacco dependence scale to directly measure substance addiction. Accordingly, we reset the questionnaire for substance addiction to speculate on the relationship between substance addiction and NSSI by measuring whether the alcohol/tobacco use frequency could predict the occurrence of NSSI (frequency of use was deemed as a relevant proxy for substance dependence).

A general questionnaire on the drinking/smoking frequency was set up for the study. The drinking frequency was categorized into the following five levels: Never (never drank), Abstinent (drank but no longer), Little (less than once a week), General (1–2 times a week), and Massive (almost every day). The smoking frequency was also categorized into five levels: Never (never smoked), Abstinent (smoked but no longer), Little (less than 10 cigarettes/day), General (11–20 cigarettes/day), and Massive (more than 20 cigarettes/day).

#### 2.2.2. Smartphone addiction scale

This study used smartphone addiction (SPA) as a quantitative criterion for non-substance addiction. Smartphones are more accessible to adolescents than alcohol and tobacco. In addition, individuals with smartphone addiction did exist in the pre-experiment with high numbers, so we set smartphone addiction as a direct marker of non-substance addiction. Combining the results of the correlation between substance addiction and non-substance addiction, we can then speculate whether the association between addiction and NSSI is present in the adolescent sample of this study.

In this study, a 22-item scale developed by Su Shuang et al. was used to measure smartphone addiction. The items were rated on a 5-point Likert scale, with 0 indicating “very unlikely” and 4 indicating “very likely.” Mean scores were used in the analysis of the results, with higher scores indicating higher levels of addiction. Based on previous studies and the results of their data, the scale developers suggest that the group with a total score of 77 or more (excluding 77) or a mean score > 3.5 be classified as “smartphone phone addiction,” the group with a total score of 66 or less (excluding 66) or a mean score < 3 be classified as “non-smartphone addiction “and the remaining group was classified as “other.” The Cronbach’s coefficient for the scale was 0.91 in the present study.

#### 2.2.3. Youth self-harm questionnaire

The questionnaire was developed by Yu (2008), in conjunction with the Intentional Self-Injury Scale developed by Graze in 2001, and was tested for reliability by adding, deleting and modifying items from the original questionnaire. This questionnaire is a self-assessment questionnaire that assesses self-injury by the formula: self-injurious behavior = number of times × severity of injury. The number of self-injuries was categorized into the following four levels: 0, 1, 2–5, 5 and more. The degree of injury to the body was assessed on 5 scales: none, mild, moderate, severe, and very severe. The Cronbach’s coefficient for the scale was 0.82 in the present study.

#### 2.2.4. Screening for genes associated with addiction

There are few data for NSSI high-throughput sequencing studies, so we searched the NCBI-GEO public database (*NCBI-GEO*) with “alcoholism [MeSH Terms] OR Alcohol addiction [ALL Fields]” and “Homo sapiens [porgn]” and “tobacco”[MeSH Terms] OR “tobacco products”[MeSH Terms] OR “Nicotiana tabacum”[Organism] OR Tobacco[All Fields]) AND (“behavior, addictive”[MeSH Terms] OR addiction[All Fields].

The R-language-based online tool GEO2R (*GEO2R*) was used to analyze and screen the differentially expressed genes (DEGs) expressed in the selected GEO dataset. Volcano maps of DEGs were visualized for expression using Sangerbox platform (30).

#### 2.2.5. Go and KEGG function enrichment analysis of DEGs

Kyoto Encyclopedia of Genes and Genomes (KEGG) pathway enrichment analysis and Gene Ontology (GO) function annotations were performed on the screened differentially expressed genes using the online analysis tool Database for Annotation, Visualization and Integrated Discovery database (DAVID version 6.8) (*Database for Annotation, Visualization and Integrated Discovery*). When screening for significant GO terms and KEGG pathways, we calculated corrected *p*-values by the EASE Score algorithm, and terms with *p* < 0.05 were considered significant.

#### 2.2.6. Construction and analysis of PPI network

Studying functional interactions between proteins may reveal mechanisms affecting disease pathogenesis or progression, therefore data screened in the STRING (Search Tool for the Retrieval of Interacting Genes/Proteins) database was used to construct a protein–protein interaction (PPI) network by setting the parameter “Minimum required interaction score: highest confidence level (0.900) and the maximum number of interaction items showing more than 50” to analyze the PPI networks. The search results were visualized using Cytoscape version 3.8.2 (31). Then, Cytoscape’s MCODE (Molecular Complex Detection) plugin was used to filter important modules of the PPI network (32). Finally, to study the significant nodes in biological networks, the study used the topological analysis method provided by CytoHubba -Maximal Clique Centrality (MCC) to rank and evaluate hub genes. From the top 10 genes, we selected the hub genes for the subsequent study (33).

#### 2.2.7. RT-qPCR experimental validation and ROC curve analysis

Using peripheral venous blood collected from patients and healthy controls, an RT-qPCR experiment was performed to validate key genes and genes with high differential expression and analyzed Receiver Operating Characteristic (ROC) curves to determine if they have the potential to become biological markers for clinical diagnosis.

Total RNA was isolated in 5 ml of blood samples using TRIZOL reagent, and the concentration of RNA was determined separately using an Implen ultra-micro spectrophotometer. Reverse transcription and RT-qPCR experiments were performed using kits from TaKaRa. Relative quantification was calculated according to the formula RQ = 2–ΔCt to verify whether there was a difference in cycle threshold (Ct) value between the target gene and the internal control (mRNA-ACTIN) compared to the controls. *p* < 0.05 was considered statistically different.

### 2.3. Statistical analyses of questionnaire

Bivariate correlation analysis was used to perform descriptive statistics and correlation analysis on the variables involved in this study, recording each variable’s mean and standard deviation and the correlation between the two variables.

Whether the NSSI differed in drinking/smoking frequency and smartphone addiction was revealed by analysis of variance (ANOVA). Paired comparisons were then used to analyze which groups of each variable had significant differences between means to verify whether individuals with high substance use frequency and individuals with smartphone addiction scored higher on the NSSI.

## 3. Results

### 3.1. Correlations among variables and analysis of variance

Statistical descriptions and correlations of all variables are presented in Table 1. Based on the results of the data, drinking/smoking frequency and smartphone addiction had a significant positive association with NSSI. After including gender and age in the correlation test of the variables, it was found that there was a significant correlation between gender and drinking/smoking frequency, smartphone addiction, and NSSI, but age was not significantly correlated with both variables. We used Independent Sample Tests to test whether there was a difference in NSSI between boys and girls. The results showed a significant difference between boys (*N* = 521, *M* = 0.017) and girls (*N* = 808, *M* = 0.055), *t* = −3.662, *p <* 0.001, specifically, girls were more likely to develop NSSI behaviors. The Chi-Square Tests allowed us to further examine the differences in drinking/smoking frequency and smartphone addiction between boys and girls. The results revealed that there was a significant difference in drinking (*χ*^2^ = 93.235, *p <* 0.001)/smoking (*χ*^2^ = 87.167, *p <* 0.001) frequency between boys and girls, except smartphone addiction (*χ*^2^ = 4.413, *p =* 0.110). Therefore, in the following ANOVA, gender was controlled as a covariate to prevent it from influencing the test of variance of main variables.

**Table 1:** Means, standard deviations, and correlations of the main study variables.

Variables	*M*	SD	1	2	3	4	5	6
1. Gender	1.610	0.488	–					
2. Age	16.510	0.566	−0.039	–				
3. Drink	1.750	1.021	−0.237^***^	0.003	–			
4. Smoke	1.160	0.525	−0.232^***^	−0.005	0.432^***^	–		
5. Smartphone	1.359	1.065	0.122^***^	0.037	0.063^*^	0.003	–	
6. NSSI	0.040	0.183	0.100^**^	−0.037	0.152^***^	0.093^**^	0.077^**^	–

Gender is a dummy variable, male is 1 and female is 2; ^*^p < 0.05, ^**^p < 0.01, ^***^p < 0.001.

The results of the ANOVA are presented in Table 2. A significant difference in NSSI between different groups of drinking/smoking frequency and smartphone addiction can be seen in the table. Through pairwise comparisons, we also found significant variability between the means of different groups for the three variables. The means of “Massive” was significantly higher than the means of “Never” (*p <* 0.001,95%CI = [0.332,1.228]), “Abstinent” (*p =* 0.036,95% CI = [−0.902,-0.030]), “Little” (*p =* 0.032,95%CI = [−0.732,-0.033]) and “General” (*p =* 0.038,95%CI = [−1.378,-0.041]) in drinking frequency; the means of “Never” was significantly lower than the means of “Abstinent” (*p <* 0.001,95%CI = [−0.634,-0.214]), “Little” (*p <* 0.001, 95%CI = [−0.936,-0.344]) in smoking frequency; the means of “SPA” was significantly higher than the means of “Non-SPA” (*p =* 0.008, 95%CI = [−1.736,-0.257]), “Other” (*p =* 0.016,95%CI = [0.180,1.734]) in smartphone addiction.

**Table 2:** Analysis of Variance (ANOVA) between variables and NSSI.

Variables	Drinking					Smoking					Smartphone addiction		
	Never	Abstinent	Little	General	Massive	Never	Abstinent	Little	General	Massive	Non-SPA	SPA	Other
*M*	−0.117	0.065	0.203	−0.219	0.499	0.033	0.091	0.137	0.044	0.000	−0.005	0.953	0.004
SD	0.477	1.211	1.536	0.000	1.702	0.165	0.291	0.298	0.099	0.000	1.001	2.004	0.786
*F*	13.177^***^					7.711^***^					3.517^*^		
*η* ^2^	0.038					0.023					0.005		

Gender was controlled as a covariate. Each level of a variable is set in order as a dummy variable from 1–5 or 1–3. M and SD are the mean and standard deviation of the different levels of the variable. The scores of non-suicidal self-injury are standardized scores. SPA referred to smartphone addiction. ^*^p < 0.05, ^***^p < 0.001.

The results in Tables 1, 2 demonstrated that the higher the drinking/smoking frequency, the higher the NSSI scores of the adolescents and the significant differences with other lower frequency groups (the “General” and “Massive” groups in smoking frequency can be ignored because of their lack of representation due to too few people). Therefore, it is reasonable to infer that as an individual’s substance use frequency increased, the NSSI scores increased as well. Since people who were addicted to substances used substances more frequently than normal individuals, the NSSI scores would continue to increase as the level of substance addiction increased. Accordingly, there was a positive correlation between substance addiction and NSSI. Besides, for smartphone addiction, the NSSI scores of people who were addicted to smartphones were significantly higher than those of non-addicted and increased as the level of addiction increased. Thus, we hypothesized that there was a positive correlation between non-substance addiction and NSSI.

In summary, both substance addiction and non-substance addiction were positively associated with NSSI, so we concluded that addiction was significantly associated with NSSI in the Chinese adolescent population in the sample of this study. All results of the questionnaire are shown in Supplementary Table S2.

### 3.2. Screening for differential genes associated with addiction

**Figure 1:**
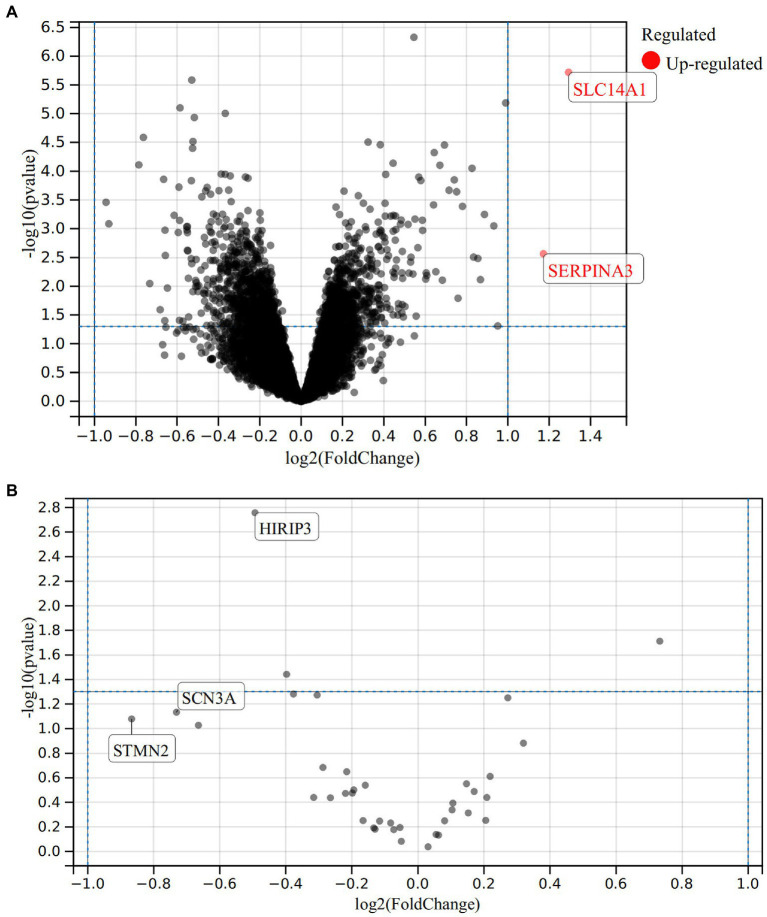
Differentially expressed genes for alcohol addiction in the GSE44456 dataset, bounded by *p* < 0.05, with red dots representing upregulated genes **(A)**; Differentially expressed genes associated with smoking addiction in the GSE20568 dataset **(B)**.

The dataset GSE44456 for alcohol addiction-related gene expression was retrieved from The Gene Expression Omnibus database. The database is based on the GPL6244 platform [HuGene-1_0-st] Affymetrix Human Gene 1.0 ST Array [transcript (gene) version] for a comparative study of gene expression in postmortem hippocampal tissue of 20 alcoholics and 19 controls (34). Analysis was performed by NCBI’s (National Center for Biotechnology Information) GEO2R online tool, in which unidentified and duplicate genes were removed. As shown in Figure 1A, 1721 DEGs were obtained by screening according to the statistical criterion of *p* < 0.05, of which 795 were up-regulated expressions and 926 were down-regulated. Data were processed using |log_2_FC| ≥ 1 as the criterion, and a total of 2 differentially expressed up-regulated genes, *SERPINA3* and *SLC14A1*, were screened for more significant expression. Analysis of the differentially expressed dataset GSE20568 for smoking addiction versus healthy controls yielded a total of 38 differentially expressed genes, and no significantly expressed genes were identified after screening by the same method (Figure 1B).

### 3.3. Go and KEGG function enrichment analysis of the DEGs

**Figure 2:**
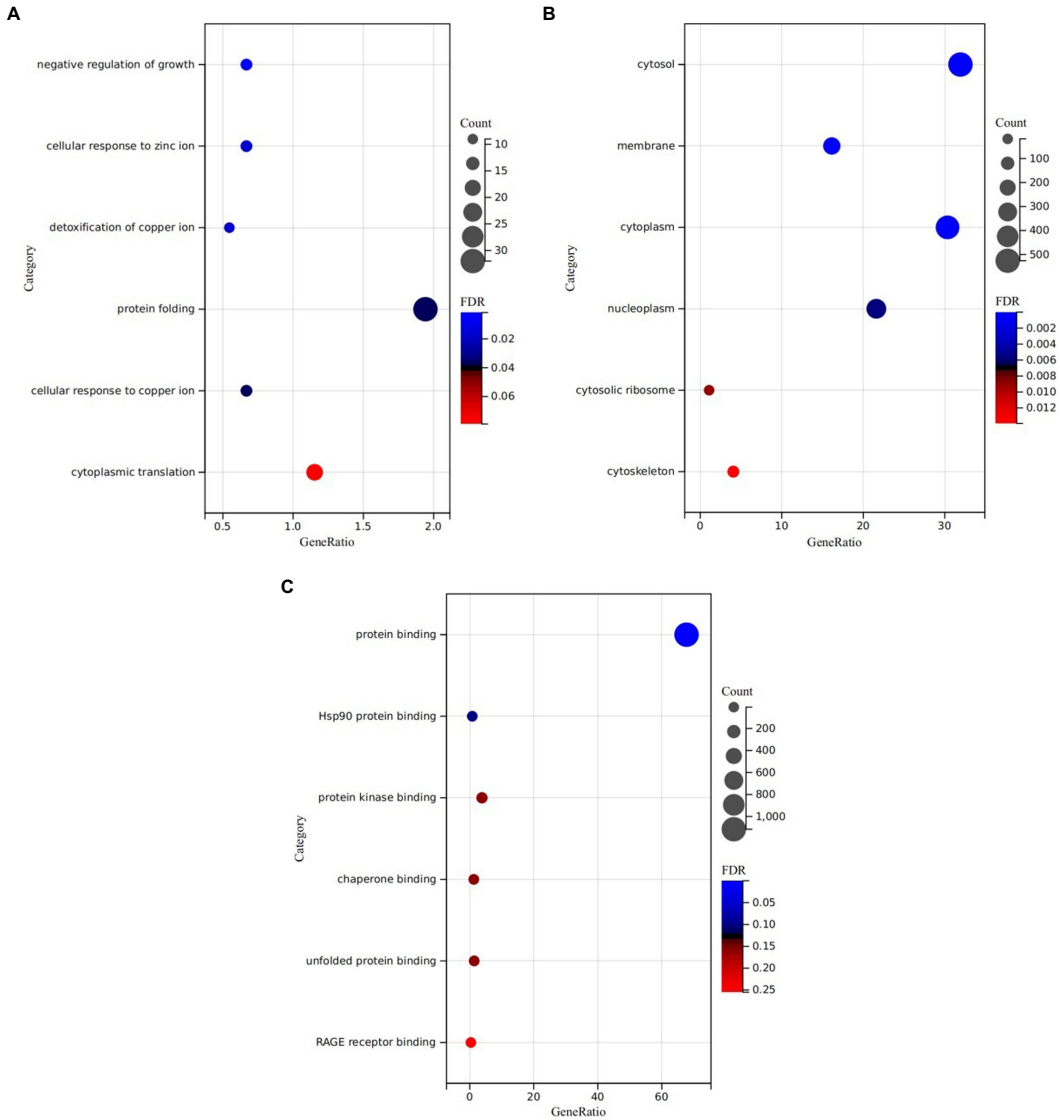
GO function enrichment analysis of differentially expressed genes: **(A)** Biological Process (BP), **(B)** Molecular Function (MF), and **(C)** Cellular Component (CC).

We analyzed the GO and KEGG pathways of alcohol addiction-related DEGs using DAVID. GO analysis showed that in Biological Process (BP), genes were mainly enriched in choline transport, signal transduction and ion transport, etc. For Cellular Component (CC) enrichment analysis, significant enrichment was observed in the cytosol, neuronal cell body and axon, etc. In the enrichment analysis of Molecular Function (MF), genes were mainly enriched in protein kinase binding, phosphatidylinositol binding, Hsp70 protein binding, and mRNA binding. The first five enrichments in the GO analysis are shown in Figure 2. The results of all GO enrichment analyses are shown in Supplementary material 1. KEGG pathway analysis revealed that alcohol addiction-related DEGs were enriched in protein kinase binding, phosphatidylinositol binding, Hsp70 protein binding and mRNA binding pathways, and all results are shown in Supplementary Figure S1.

### 3.4. Construction and analysis of PPI network

**Figure 3:**
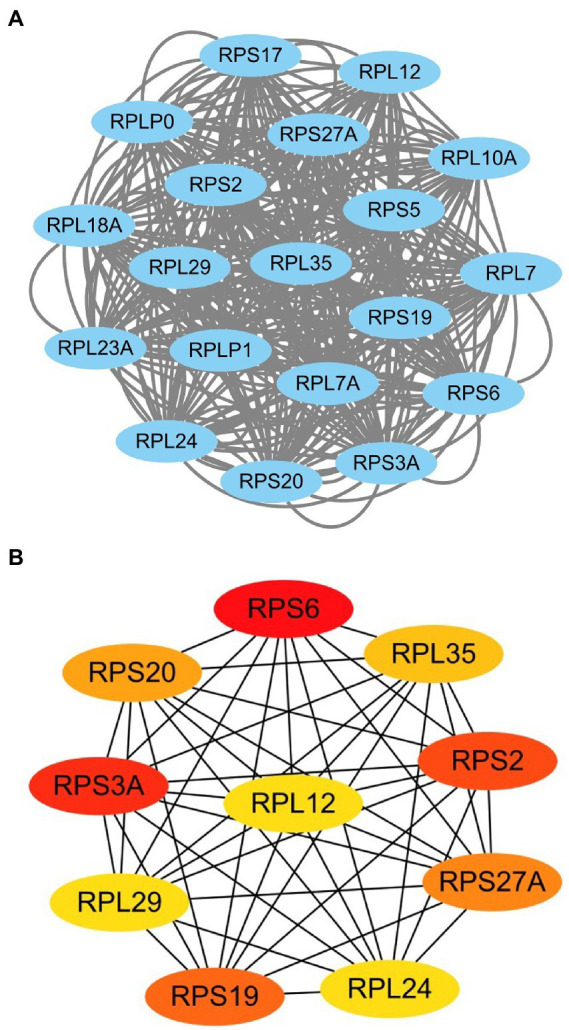
Top scoring important modules in the PPI network **(A)** and the first 10 central genes **(B)**. Node color shows rank (the darker the color, the higher the rank).

We imported 1721 alcohol addiction-related DEGs into the STRING database, a total of 10 cluster modules were obtained from the PPI network using MCODE screen modules. As shown in Figure 3A, the highest-scoring module (Score: 19) consists of 19 nodes and 342 edges. The first 10 genes were identified as hub genes by the MCC method as shown in Figure 3B.

We selected the two most significantly differentially expressed genes *SERPINA3* (log_2_FC = 1.17, *p* < 0.01) and *SLC14A1* (log_2_FC = 1.29, *p* < 0.001) screened from the GEO database and the top two ranked hub genes *RPS6* (log_2_FC = 0.14, *p* < 0.05) and *RPS3A* (log_2_FC = 0.16, *p* < 0.05) from the highest scoring modules of the PPI network analysis for the next step of validation experiments in clinical samples.

### 3.5. RT-qPCR experimental validation and ROC curve analysis

A total of 48 clinical study subjects were included in this study. Of the 24 NSSI patients, 21 were female and 3 were male, aged 15–22 (16.0 ± 2.5) years. Of the 24 healthy controls, 12 were female and 12 were male, aged 16–68 (31.6 ± 11.3) years. The results of the Chi-square Tests for age and gender in NSSI patients and healthy controls showed that gender (*χ*^2^ = 9.375, *p =* 0.005) and age (*t* = 6.570, *p =* 0.000) were significantly different between the two groups. The characteristics of the clinical blood samples are shown in Supplementary Table S3.

**Figure 4:**
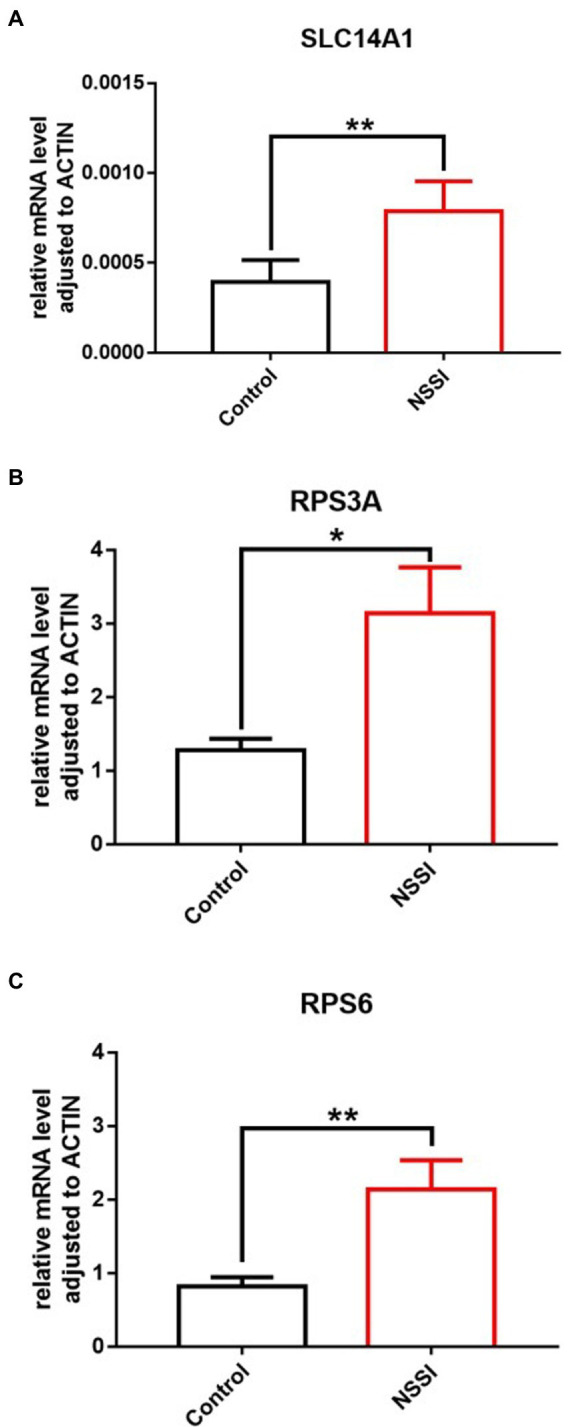
Expression levels of *SLC14A1*/*RPS6*/*RPS3A* in peripheral blood of NSSI patient group and healthy control group: **(A)** *SLC14A1*, **(B)** *RPS3A*, and **(C)** *RPS6*. *p<0.05, **p<0.01.

**Figure 5:**
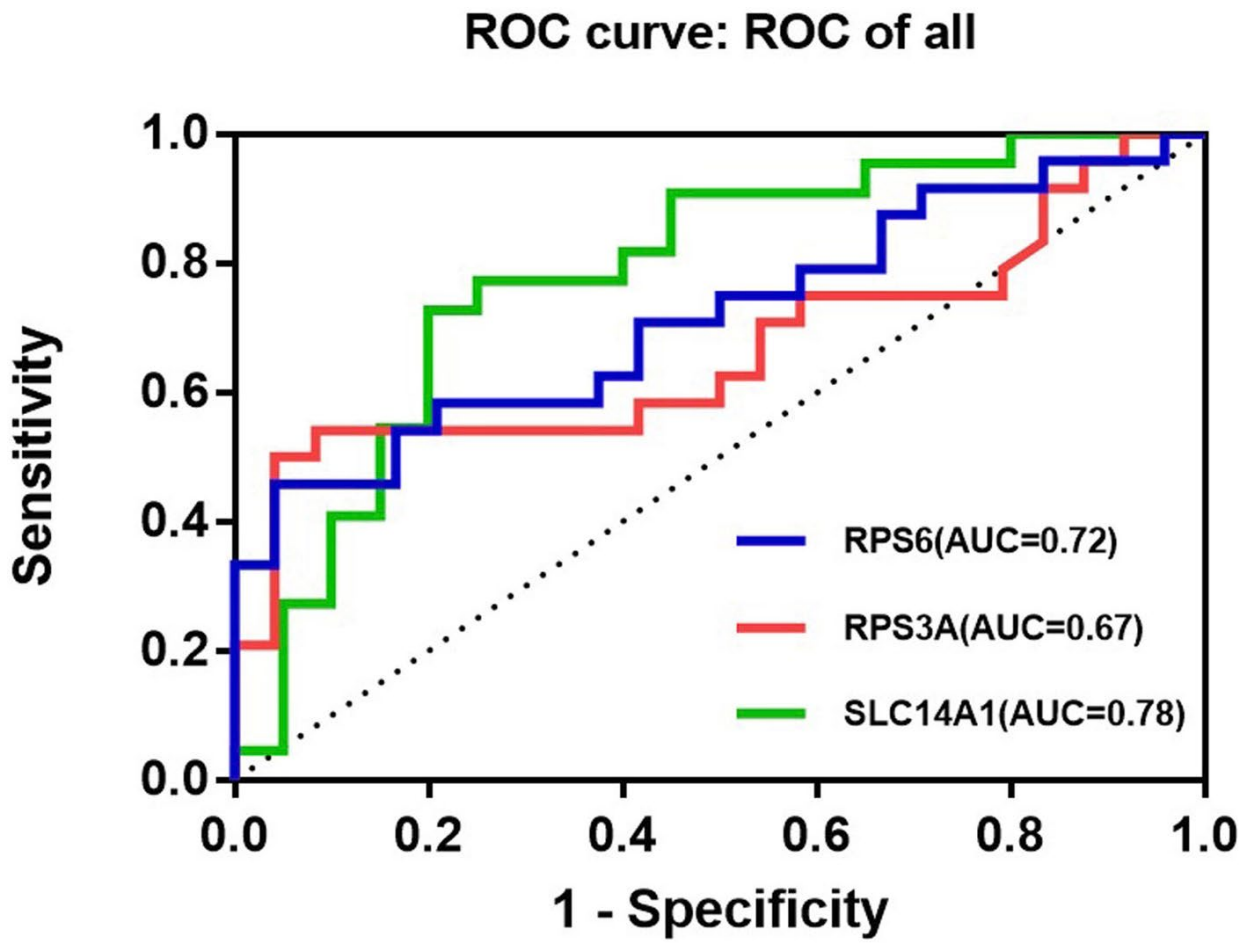
Receiver Operating Characteristic (ROC) curves of *SLC14A1*/ *RPS6*/*RPS3A*.

In the RT-qPCR experiment, the relative quantitative analysis of *SERPINA3* (Serpin peptidase inhibitor, clade A (alpha-1-antiproteinase, antitrypsin), member 3), *SLC14A1* (Solute Carrier Family 14 Member 1), *RPS6* (Ribosomal Protein S6) and *RPS3A* (Ribosomal Protein S3A) was performed using ACTIN as the internal reference gene. The results revealed that the expression levels of *SLC14A1* (*p* = 0.0014), *RPS6* (*p* = 0.0092) and *RPS3A* (*p* = 0.0489) were significantly higher in the peripheral blood of NSSI patients than in control blood samples, and the differences were statistically significant (Figure 4). Supplementary Table S4 illustrated the results of the relative quantitative analysis. Subsequently, we plotted ROC curves for the screened genes with significant differences in *SLC14A1*, *RPS6* and *RPS3A*. The results of ROC curves for different genes are marked with different colors in Figure 5. Based on the results, we found that the AUC of *SLC14A1* was 0.78, the specificity was 72.7%, and the sensitivity was 80.0%; the AUC of *RPS6* was 0.72, the specificity was 45.8%, and the sensitivity was 95.8%; the AUC of *RPS3A* was 0.67, specificity was 54.2%, and sensitivity was 91.7% for *RPS3A*.

## 4. Discussion

Non-suicidal self-injury is the intentional injury of bodily tissues of the self in any way that is not accompanied by suicidal intent (35). It has a high prevalence and potential for future suicidal behavior in adolescent populations worldwide (36, 37), and in recent studies it has been found that repetitive non-suicidal self-injury may develop addictive behaviors leading to an increase in frequency and intensity (38, 39), with serious implications for adolescent health and life safety. The present study was conducted to verify the significant correlation between non-suicidal self-injury and addiction in a Chinese adolescent population combined with molecular biology experiments to explore clear biomarkers to demonstrate the association between non-suicidal self-injury and addiction.

We first investigated substance addiction (alcohol/tobacco), non-substance addiction (smartphone addiction) and non-suicidal self-injurious behavior using questionnaires among adolescents aged 16–18 years in a region of China. The results of the questionnaire showed that the proportion of non-suicidal self-injury in the adolescent group selected for this study was 12.2%, which was similar to the results of previous studies (12). Correlation analysis showed a significant positive association between drinking/smoking frequency, smartphone addiction and NSSI. Analysis of variance (ANOVA) with gender as a covariate suggested that the NSSI scores of adolescents in the high substance use group were significantly higher than in the lower frequency groups; adolescents in the smartphone addiction group had significantly higher NSSI scores than those in the non-addiction group. It can be inferred that both substance addiction and non-substance addiction have a significant positive association with NSSI. From these statistically calculated results, we suggest that a significant association between addiction and non-suicidal self-injury exists in the Chinese adolescent population.

Based on the theories derived from the questionnaire and statistical analysis, the study chose to use bioinformatics techniques and molecular biology experiments for further experimental validation. We did not obtain significant expression in tobacco addiction-related differential genes by bioinformatics analysis. However, four genes, SERPINA3, SLC14A1, RPS6 and RPS3A, were obtained among the alcohol addiction-related differential genes. Next, we selected these four genes as the target genes for molecular biology experiments. Validation analysis with the help of RT-qPCR experiment revealed that among the four target genes, *SLC14A1*, *RPS6* and *RPS3A* were significantly differentially expressed, and the expression levels of these three genes were significantly higher in the venous peripheral blood of NSSI patients than in normal subjects.

Solute Carrier Family 14 Member 1 (*SLC14A1*) belongs to a family of proteins that facilitate the transport of urea. It is more widely distributed in human tissues than other factors in the family (40). *SLC14A1* is found to be a diagnostic marker in patients with Progressive Supranuclear Palsy (PSP) in studies of neurological diseases (41). In addition to this, based on evidence from Aimée C. Jones, *SLC14A1* is involved in regulating the inflammation of microglial cells and neuron-like cells and is associated with neurodegenerative disease pathogenesis (42); Serpin peptidase inhibitor, clade A (alpha-1-antiproteinase, antitrypsin), member 3 (*SERPINA3*) is a secreted serine protease inhibitor. It is involved in regulating the development of glioblastoma and colon cancer (43, 44); Ribosomal Protein S6 (*RPS6*) is an important protein component of the 40S small subunit and was the first ribosomal protein found to be phosphorylated (45). It has the potential as a molecular marker of ovarian cancer (46); Ribosomal Protein S3A *(RPS3A)* is a member of the S3AE family of ribosomal proteins, and also a component of the 40S subunit. RPS3A has been less studied in neuropsychiatric disorders and has only been reported in AD (47). The regulatory roles of these genes in the pathogenesis of tumors as well as other diseases have been revealed more intensively, but studies on their mechanisms of action in psychiatric disorders are still scarce.

Subsequently, ROC curves were used to analyze whether these genes have the potential to become biological markers for the clinical diagnosis of NSSI. As indicators for diagnosing the disease, AUC and specificity are considered as the primary reference values. The two aspects of the ROC curve are combined to determine that the *SLC14A1* gene may be of high value for the clinical diagnosis of NSSI, with a higher value of AUC (0.78) and specificity (72.7%) than the other two genes. The AUC value of *RPS6* (0.72) is higher than that of *RPS3A* (0.67), indicating that its overall diagnostic value is slightly higher than that of *RPS3A*. However, the specificity value of *RPS6* (45.8%) differed significantly from that of *RPS3A* (54.2%), which is less sensitive than *RPS3A* for disease diagnosis. In conclusion, *SLC14A1*, *RPS6* and *RPS3A* can potentially become biological markers for clinical diagnosis of NSSI, with differences in the value and specificity of different genes.

It is worth noting that differential expression of *SERPINA3* was not verified in clinical specimens in molecular biology experiments, probably because the GEO database screens for differential genes from brain samples, but clinical collections are from peripheral blood samples of patients. The presence of the blood–brain barrier may affect the differential expression of genes in the central and peripheral nervous systems. In addition, differences in the statistical and computational methods of the databases in which bioinformatics analysis was performed may lead to false positive prediction results.

The gender difference in NSSI patients recruited for this study showed that there were far fewer males than females. Based on previous studies on the effect of gender on NSSI, it was found that there is a large gender difference in the prevalence of NSSI between men and women, specifically, men are much less likely than women (48). This led to a gender bias in the recruited patients. On the other hand, the age difference between the patient group and the healthy control group in this study showed significant results, resulting from the fact that NSSI showed a higher prevalence in the adolescent population than in the adult population (49, 50). Therefore, a greater number of patients randomly recruited in this study were adolescents.

Non-suicidal self-injury, as self-injurious behavior with the primary attempt to relieve environmental stress or regulate emotions, is a possible protective mechanism for depression and even suicide (Gratz and Chapman, 2007; Morales, 2013; Peel-Wainwright et al., 2021). However, researches by Sarah and Blasco-Fontecilla et al. suggest an association between NSSI and addiction (Hilario et al., 2016; (24)), likely leading to overuse of self-injurious behaviors to avoid the effects of negative information. If environmental factors continue to worsen or emotion regulation strategies fail, self-injurious behavior will be repeated over and over in response to the adverse event (Zetterqvist and Maria, 2015). The threshold for obtaining pleasure after self-harm also increases over time, meaning that individuals need to increase the frequency or degree of self-harm to obtain the same relief as before (13). The mechanism of this behavior is very similar to addiction and may result in eventual suicidal ideation leading to the complete liberation of the individual. We observed this behavior and predicted that its severe consequences may threaten the lives of adolescents. Therefore, we hope to use the existing research to reveal the association between addiction and NSSI from a more fundamental perspective as a way to alert adults to pay more attention to self-injurious behaviors in adolescents.

## 5. Limitation

Firstly, the questionnaire section of this study relied solely on subjective self-reports which could induce bias. Future research can also rely on multiple informants (e.g., parent reports and peer reports), which will afford more rigorous survey data and enrollment criteria. Secondly, the samples in the current study were all adolescents from one region of Yunnan Province, which may affect the generalizability of the results, and future studies could select an adequate number of samples from adolescents in different regions. In addition, the number of venous peripheral blood samples collected from NSSI patients as well as healthy controls in the study was limited. In the next step, we will consider expanding the sample size to confirm the differential expression of *SLC14A1*, *RPS6* and *RPS3A* in more patient specimens. It is worth noting that we only found addiction genes with differential expression in NSSI patients in the present study, confirming that there is indeed a clear association between addiction and non-suicidal self-injury, but we did not delve into how exactly the mechanism of action of these genes on non-suicidal self-injury. Then we hope that we can focus on this direction in future research and reveal the mechanism of action of addiction genes inducing non-suicidal self-injury by some technical means.

## 6. Conclusion

1. The significant association between addiction and non-suicidal self-injury exists in the Chinese adolescent population.
2. Addiction-related genes *SLC14A1*, *RPS6* and *RPS3A* are differentially expressed in adolescents with non-suicidal self-injury. The present study confirms the association of addiction with non-suicidal self-injury from a molecular biological perspective. The genes *SLC14A1*, *RPS6* and *RPS3A* have the potential to become biological markers for clinical diagnosis of non-suicidal self-injury.

## Data availability statement

Publicly available datasets were analyzed in this study. This data can be found here: GSE44456.

## Ethics statement

The studies involving human participants were reviewed and approved by Ethical Committee of Kunming Medical University (2022kmykdx6f47). The study was conducted according to the guidelines of the Declaration of Helsinki. Written informed consent to participate in this study was provided by the participants’ legal guardian/next of kin.

## Author contributions

ZG and YL: conceptualization, formal analysis, and writing—original draft preparation. YoZ and ZT: methodology, resources, and supervision. ZG and CW: validation. SL and WW: investigation. LY and CW: data curation. XY and YuZ: writing—review and editing and project administration. YoZ, YuZ, XY, and WW: funding acquisition. All authors contributed to the article and approved the submitted version.

## Funding

The research was financed by the National Natural Science Foundation of China (grant numbers 82260276, 81960254 and 82060257), Joint special fund of Applied Fundamental Research of Kunming Medical University granted by Science and Technology Office of Yunnan (grant numbers 202101AY070001-196, and 202201AY070001-181), Yunnan health training project of high-level talents (grant no. L-2017021), Yunnan Provincial Department of Education Research Fund (grant number 2022Y196), and Teaching and Reform Project of the Steering Committee for Teaching Psychology in Higher Education Institutions of the Ministry of Education (grant number 20221002). The funders had a role in study design, but they had no role in data collection and analysis, decision to publish, and preparation of the manuscript.

## Conflict of interest

The authors declare that the research was conducted in the absence of any commercial or financial relationships that could be construed as a potential conflict of interest.

## Publisher’s note

All claims expressed in this article are solely those of the authors and do not necessarily represent those of their affiliated organizations, or those of the publisher, the editors and the reviewers. Any product that may be evaluated in this article, or claim that may be made by its manufacturer, is not guaranteed or endorsed by the publisher.
