# Supplementary material for: Exploring the association of addiction-related genetic factors with non-suicidal self-injury in adolescents
Source: Front Psychiatry. 2023 Mar 31;14:1126615. doi: 10.3389/fpsyt.2023.1126615 (PMC10102595; doi:10.3389/fpsyt.2023.1126615)
Supplement: Supplementary file 5 [file Data_Sheet_1.docx]

Supplementary Material 1 - Data for GO Analysis

| GO ID | Term | P-value | Genes |
| --- | --- | --- | --- |
| BP |  |  |  |
| GO:0045926 | negative regulation of growth | 0.00000 | ING4, MT2A, MT1A, PNPT1, IGFBP5, MT1M, MT1G, MT1X, MT1B, HIF1A, MT1E |
| GO:0071294 | cellular response to zinc ion | 0.00001 | GLRA1, MT1DP, MT2A, MT1A, CREB1, MT1M, MT1G, MT1X, MT1B, MT1HL1, MT1E |
| GO:0010273 | detoxification of copper ion | 0.00001 | MT1DP, MT2A, MT1A, MT1M, MT1G, MT1X, MT1B, MT1HL1, MT1E |
| GO:0006457 | protein folding | 0.00003 | HSP90AA2P, ERO1B, UBXN1, HSP90AB4P, NGLY1, HSP90AA6P, GNAI1, HSP90B1, DNAJB1, DNAJB6, BAG2, BAG3, DNAJC21, CCT5, CCT4, RANBP2, CCT2, HSPA8, MKKS, HSP90AA1, PTGES3, AHSA1, HSPE1, PDIA6, PDIA4, DNAJA1, GNB2, DNAJB11, TCP1, FKBP4, PPIL3, PPID |
| GO:0071280 | cellular response to copper ion | 0.00004 | APP, MT1DP, MT2A, MT1A, HSF1, MT1M, MT1G, MT1X, MT1B, MT1HL1, MT1E |
| GO:0002181 | cytoplasmic translation | 0.00011 | RPLP1, RPS5, RPL12, RPS6, RPLP0, TMA7, RPL23A, RPS3A, RPL10A, RPL7, RPL7A, RPS17, RPS19, RPL24, RPL35, RPS20, RPS2, RPL29, RPS27A |
| GO:0006695 | cholesterol biosynthetic process | 0.00012 | SQLE, IDI1, CYB5R3, HMGCS1, GGPS1, INSIG2, INSIG1, PMVK, MSMO1, DHCR24, HMGCR, ACAT2 |
| GO:0006882 | cellular zinc ion homeostasis | 0.00013 | MT1DP, MT2A, MT1A, MT1M, SLC39A6, SLC39A10, MT1G, MT1X, MT1B, MT1HL1, MT1E |
| GO:0043312 | neutrophil degranulation | 0.00016 | SERPINA3, PYGB, CDA, ATP8A1, SLC44A2, HBB, PYGL, SLC2A5, HEBP2, LGALS3, GLIPR1, PNP, FTH1, LAMP2, COTL1, ENPP4, GOLGA7, CEP290, CCT2, HSP90AA1, CD93, ANXA2, ANXA3, SLC11A1, ADAM10, MCEMP1, OLFM4, APRT, CRISPLD2, TMBIM1, PECAM1, ADAM8, S100A9, S100A8, DNASE1L1, TMEM63A, STXBP3, MOSPD2, MGST1, FPR1, PSEN1, CFP, PLD1, CYB5R3, CXCR1, NEU1, CXCR2, S100A12, STOM, CD59, METTL7A, HSPA8, MGAM, GCA, DNAJC13, LILRB3, CPPED1, TSPAN14, IMPDH1, QPCT, SLCO4C1 |
| GO:1900034 | regulation of cellular response to heat | 0.00022 | RANBP2, HSPA8, HSP90AA1, HSPA1L, HSBP1, PTGES3, HIKESHI, AAAS, DNAJB1, HSPH1, DNAJB6, BAG2, BAG3, HSF1, CHORDC1, ATM, FKBP4 |
| GO:0015871 | choline transport | 0.00026 | SLC44A5, SLC44A3, SEC14L1, SLC44A1, SLC44A2, PSEN1 |
| GO:0050821 | protein stabilization | 0.00033 | HIP1, HSP90AB4P, CCNH, CSN3, USP19, RTN4, C10ORF90, NCLN, RASSF2, BAG2, BAG3, CHEK2, LAMP2, DVL1, PIM1, GOLGA7, CCT5, CCT4, CCT2, HSP90AA1, COG7, PTGES3, COG3, HPS4, IGF1, A1CF, CREB1, PRKRA, TCP1, MDM4, NAA15 |
| GO:0006614 | SRP-dependent cotranslational protein targeting to membrane | 0.00046 | RPLP1, RPS5, RPL12, RPS6, RPLP0, RPL23A, RPS3A, RPL10A, RPL7, RPL7A, RPS17, RPS19, RPL24, RPL35, RPS20, RPS2, RPL29, RPS27A |
| GO:0042634 | regulation of hair cycle | 0.00047 | FA2H, PER1, EPS8L3, CLOCK, ARNTL |
| GO:0019216 | regulation of lipid metabolic process | 0.00053 | SERPINA3, IDI1, GGPS1, HMGCS1, NFYB, NR1D2, ELOVL6, HMGCR, DNAJC15, ACACB, ACACA, SQLE, TBL1XR1, SCD, ID2, PMVK, PPARD |
| GO:0019083 | viral transcription | 0.00080 | RANBP2, RPLP1, RPS5, RPL12, RPS6, RPLP0, RPL23A, RPS3A, RPL10A, AAAS, RPL7, RPL7A, RPS17, RPS19, RPL24, RPL35, RPS20, RPS2, RPL29, RPS27A |
| GO:0006986 | response to unfolded protein | 0.00082 | HSPA8, HSP90AA1, HSPA1L, FICD, HSPA4, HSPB7, HSPE1, DNAJA1, DNAJB1, HSPH1, DNAJB5, JKAMP, STT3B |
| GO:0071276 | cellular response to cadmium ion | 0.00088 | MT1DP, MT2A, MT1A, HSF1, MT1M, AKT1, MT1G, MT1X, MT1B, MT1HL1, MT1E |
| GO:0007165 | signal transduction | 0.00109 | RERGL, CNTFR, NR3C1, GLDN, CRHBP, CCND3, RASSF2, ALCAM, GRB14, DPYSL5, RASSF4, RASSF5, TNFSF10, AKT1, SOX9, MAP3K6, PDE8A, SH3GL1, IGFBP1, MAP2K3, IL4R, IGFBP5, THOC1, OLFM4, GTPBP1, MOB3B, TOM1L2, ZFYVE16, S100A6, PRKD1, STAM2, CLOCK, PLPP2, CDC42SE1, RASL12, COPA, PXN, FPR1, GPR85, STK39, DAPP1, TNFRSF11B, HIF1A, RACGAP1, SMAD1, STARD13, DTNA, TSLP, OSGIN1, STAM, IGF1, NR4A2, COPS3, NR4A1, ARHGAP31, GNB2, PPP1R1B, PDE3A, NOSTRIN, CSPG5, RADIL, ATM, OR6A2, MYD88, HBS1L, ADM, VLDLR, PIK3CB, ARHGAP6, IMPA2, PPP2R5E, KIF13B, RAC2, PRKG2, TIMP1, CLIC1, PRMT2, ARAP3, TNFRSF1A, GREM1, NCR1, CREB1, RABEP1, NRG4, IL1RAPL1, PECAM1, RARB, PPP1R12B, ZP3, MET, PPP1R12C, TRIM55, PLPPR1, INPP1, AKAP8, PRKX, GNA14, GLRA1, IL1RL1, MTA1, RASD1, ERBB3, SNX27, GNG5, ERBB2, KLRC4-KLRK1, CXCR2, PDE6B, GABRE, IL31, CMTM5, PTGES3, ERAP1, STAT3, RIT2, RPS6KB1, DLC1, GPR149, CMTM2, PKN1, PDE7A, AGRN, F2RL3 |
| GO:0000082 | G1/S transition of mitotic cell cycle | 0.00140 | RB1, PLK3, USP37, CUL5, CCNH, RPS6, CUL1, CCNE2, CCND1, RPS6KB1, MYC, ID4, EIF4EBP1, SKP2, CRLF3 |
| GO:0032869 | cellular response to insulin stimulus | 0.00165 | RB1, C2CD5, INSIG2, INSIG1, HDAC9, FOXO1, APRT, GHR, EEF2K, RPS6KB1, MYC, RAB13, PNPLA3, AKT1, TRIB3, CPEB2, FBP1, RAB8A, SLC29A2 |
| GO:0006811 | ion transport | 0.00170 | SLC24A2, SLC36A1, SLC12A2, SLC26A2, SLC20A2, PLLP, CP, SLC1A7, P2RX7, SLC8A3, GLRA1, SLC22A15, SLCO4A1, SLC9A8, SLCO2A1, SLCO1A2, LRRC8D, LRRC8A, SLC17A8, KCNN3, PLP2, SLC12A6 |
| GO:0016126 | sterol biosynthetic process | 0.00172 | CYB5R2, SQLE, INSIG2, INSIG1, PMVK, MSMO1, HMGCR |
| GO:0014065 | phosphatidylinositol 3-kinase signaling | 0.00198 | EDN1, ERBB3, PLEKHA1, ERBB2, KBTBD2, AKT1, IGF1, PIK3CB, PEAR1, PIK3C2B |
| GO:0031929 | TOR signaling | 0.00314 | CCDC88A, RPS6KB1, RPS6, EIF4EBP1, EIF4EBP2, AKT1, FNIP1 |
| GO:0034109 | homotypic cell-cell adhesion | 0.00324 | CXADR, MEGF10, MEGF11, CEACAM5, CD99 |
| GO:2000074 | regulation of type B pancreatic cell development | 0.00406 | AKT1, CLOCK, FOXO1, ARNTL |
| GO:0043065 | positive regulation of apoptotic process | 0.00407 | APP, ADM, OMA1, BCL10, PSEN1, FOXO1, IFIT2, CASP9, ING4, ING3, RASSF2, ADAMTSL4, TNFSF10, ARL6IP5, AKT1, SLIT2, SUDS3, RPS27A, TGM2, ZBP1, STPG1, KLF11, TNFRSF12A, PRMT2, TGFB3, GADD45A, ZBTB16, IGFBP3, ARHGEF17, RPS6, OSGIN1, ADAM10, DUSP6, GADD45G, DNAJA1, NR4A1, IL6, CREB1, BCL6, STK17A, TXNIP, RARB, ATM, ARHGEF2, PPID |
| GO:0006915 | apoptotic process | 0.00416 | HIP1, AHR, HIF3A, NR3C1, CASP9, ING4, ADAMTSL4, RASSF5, TNFSF10, PIM1, DPF2, ZBP1, SRGN, KLF11, PDCD6IP, TNFRSF12A, IGFBP3, THOC1, KREMEN1, NME3, TNFRSF1A, GREM1, RABEP1, RRAGC, CEACAM5, DDIT4, G2E3, ELMO1, PRKD1, S100A9, S100A8, BIRC2, PPID, PPARD, SHC4, BIRC8, MTFP1, PRUNE2, SLC5A11, CUL1, TNFRSF11B, PSEN1, BCL10, FOXO1, RTN4, FXR1, SUDS3, RNF130, PLK3, TIGAR, TIA1, GADD45A, ZBTB16, DHCR24, GADD45G, NR4A1, ITCH, RPS6KB1, STK17A, DLC1, GJB6, CLPTM1L, XAF1, MYD88 |
| GO:0002523 | leukocyte migration involved in inflammatory response | 0.00422 | ADAM8, S100A9, TRIM55, RTN4, S100A8, JAM3 |
| GO:0071260 | cellular response to mechanical stimulus | 0.00425 | MAP3K1, GADD45A, ITGA2, SLC2A1, BCL10, TNFRSF1A, BAG3, PIEZO2, AKT1, PIEZO1, SOX9, SLC38A2, MYD88, KCNJ2, PLEC |
| GO:0006486 | protein glycosylation | 0.00432 | GALNT7, ST6GALNAC1, ALG8, COG7, POGLUT1, GALNT15, COG3, B3GALT4, PSEN1, B3GNT9, EXTL2, NPC1, MAN2A2, MAN2A1, ST3GAL5, TRIP11, B3GNT2, ST3GAL6, MAN1B1, UGGT1 |
| GO:0008283 | cell proliferation | 0.00478 | USPL1, PTGES3, ITGA2, PAK1IP1, STAT3, ESRRB, CUL1, LIG4, LRP2, IGF1, USP28, LIPA, BYSL, CKS1B, TBCK, IL18RAP, MYC, AKT1, POLR3G, NELFB, CSPG4, PPARD |
| GO:0000184 | nuclear-transcribed mRNA catabolic process, nonsense-mediated decay | 0.00481 | RPLP1, RPS5, RPL12, RPS6, RPLP0, RPL23A, RPS3A, PPP2R2A, RPL10A, RPL7, RPL7A, RPS17, RPS19, RPL24, RPL35, RPS20, RPS2, RPL29, RPS27A |
| GO:0050729 | positive regulation of inflammatory response | 0.00540 | ZBP1, APP, CEBPB, FEM1A, TSLP, ITGA2, DEFB114, TNFRSF1A, IL1RL1, HYAL2, NAPEPLD, S100A12, ZP3, CLOCK, S100A9, S100A8, TGM2 |
| GO:0048661 | positive regulation of smooth muscle cell proliferation | 0.00541 | EDN1, ITGA2, HMGCR, IGF1, IL6, RPS6KB1, MYC, ID2, AKT1, SKP2, IL6R, MYD88, TGM2 |
| GO:0000122 | negative regulation of transcription from RNA polymerase II promoter | 0.00657 | RB1, APP, ZNF131, THRA, MAGEB16, ATN1, HMGB2, HNRNPU, CTCF, RBPJ, NR3C1, YBX3, TRO, CCND3, MAGEA8, CCND1, MYC, ZNF724, DPF2, ZNF404, SOX9, ZNF202, TLE4, KLF11, EDN1, ZHX3, OLIG3, EED, HSBP1, OSR1, USP3, ZNF10, DICER1, ETV5, MED25, TBL1XR1, HAND1, RFX5, TXNIP, RARB, LCOR, HCLS1, TRIB3, NUPR2, FBP1, PPID, PPARD, URI1, CEBPB, DNMT1, HDAC2, SATB1, GLIS3, ZBTB1, PSEN1, HDAC9, FOXO1, HDAC7, DNAJB1, FOXS1, MNT, MTA1, DEAF1, HSF1, HSF4, SMYD2, PCGF1, SUDS3, FNIP1, RPS27A, TCF7L2, PLK3, SEMA4D, ZBTB14, GADD45A, ZBTB16, PLK1, STAT3, NR1D2, ZNF77, SORBS3, FAM220A, NR4A2, HEYL, PER1, DLG1, BCL6, ID2, CNOT2, ZNF219, HNRNPA2B1, ID4, ZNF536, ZNF217, MDM4, MXD4 |
| GO:0050830 | defense response to Gram-positive bacterium | 0.00699 | GBP6, APP, DEFA6, DEFB114, HMGB2, ADM, MR1, P2RX7, IL6, ADAM17, DMBT1, KLRC4-KLRK1, ZG16, VIP, TAC1, IL6R, GBP4, MYD88 |
| GO:0051216 | cartilage development | 0.00727 | BBS2, SMAD1, EDN1, MKKS, HYAL2, ZBTB16, ITGB8, TRIP11, TIMP1, SOX9, HIF1A, MATN3 |
| GO:0050919 | negative chemotaxis | 0.00744 | SEMA6B, DPP4, FLRT2, SEMA4D, SEMA4B, SEMA6D, SEMA4C, SEMA3E, SLIT2 |
| GO:0015734 | taurine transport | 0.00766 | SLC36A1, LRRC8C, LRRC8D, LRRC8A |
| GO:0032496 | response to lipopolysaccharide | 0.00811 | GGT5, EDN1, HDAC2, SPARC, CNP, SLC11A1, MGST1, HMGB2, ADM, IRAK3, SOD2, P2RX7, CASP9, NR4A1, ADAM17, DUSP10, RPS6KB1, GJB6, TAC1, S100A9, S100A8, TIMP4 |
| GO:0001975 | response to amphetamine | 0.00875 | NR4A2, RANBP2, NR4A1, EDN1, HDAC2, PPP1R1B, HDAC9, DRD4, ICAM1 |
| GO:0006629 | lipid metabolic process | 0.00998 | MTMR2, LRP2, OMA1, COMT, LIPA, ACAT2, FADS3, APOL4, PLAGL2, CEPT1, ZNF202, SLC16A1, HMGCS1, CHKA, ADHFE1, SLC16A11, EPHX3, FMO5, KBTBD2, PDE3A, FAR1, PLIN1, ANGPTL4, LRP10, BCAT2, PPARD, PLA1A |
| GO:0019221 | cytokine-mediated signaling pathway | 0.01014 | CNTFR, CSF3, CEBPD, MAOA, FPR1, PIK3CB, HIF1A, FOXO1, HSP90B1, ICAM1, GHR, SOCS3, CCND1, MYC, PIM1, AKT1, TIMP1, RPS27A, IL6R, TGM2, STAT5A, HSPA8, HSP90AA1, IL31, IL4R, IFNB1, IL1R1, STAT3, IRAK3, IFNLR1, LILRB3, OSMR, IL17RC, TNFRSF1A, IL6, BCL6, FSCN1, PLP2 |
| GO:0061077 | chaperone-mediated protein folding | 0.01021 | CCT2, DNAJB6, TCP1, CHORDC1, FKBP4, CCT5, PPID, PDIA4, CCT4 |
| GO:0045821 | positive regulation of glycolytic process | 0.01030 | P2RX7, APP, MYC, ESRRB, IGF1, PSEN1, HIF1A |
| GO:0050829 | defense response to Gram-negative bacterium | 0.01064 | APP, DEFA6, TSLP, SLC11A1, DEFB114, HMGB2, ADM, MR1, IL6, RPS19, DMBT1, PRKD1, VIP, TAC1, IL6R |
| GO:0071456 | cellular response to hypoxia | 0.01096 | TIGAR, EDN1, HILPDA, HP1BP3, HIF1A, VASN, RTN4, ICAM1, SLC8A3, CCNA2, RGCC, MYC, LMNA, EIF4EBP1, AKT1, MDM4, ADAM8, CPEB2, PPARD |
| GO:1990830 | cellular response to leukemia inhibitory factor | 0.01138 | TLE4, NFYB, SPRY4, SMARCA5, HNRNPU, CACYBP, ICAM1, SOCS3, CREB1, RNF125, NR5A2, SPRY2, B3GNT2, SRSF3, SRSF7, BCAT2 |
| GO:0045737 | positive regulation of cyclin-dependent protein serine/threonine kinase activity | 0.01153 | CCND3, ADAM17, CCNK, RGCC, CCND1, PIM1, AKT1, CKS1B |
| GO:0006413 | translational initiation | 0.01182 | RPLP1, RPS5, RPL12, RPS6, RPLP0, RPL23A, RPS3A, RPL10A, RPL7, RPL7A, RPS17, RPS19, RPL24, RPL35, RPS20, RPS2, RPL29, RPS27A, EIF4G2 |
| GO:0043491 | protein kinase B signaling | 0.01185 | P2RY12, SETX, PLK3, RPS6KB1, AKT1, SOX9, IGF1, PEAR1, PIK3C2B |
| GO:0045892 | negative regulation of transcription, DNA-templated | 0.01219 | RB1, NDUFA13, THRA, GMNN, HMGB2, CTCF, AHR, RBPJ, NR3C1, ING4, LBH, DPF2, NELFB, SOX9, KLF11, TSC22D4, ZHX3, FOXD1, PRMT2, EED, ZBTB38, GREM1, HAND1, TRIB3, ZP3, CLOCK, TRIM11, PPARD, ZNF397, HDAC2, CEBPD, PBXIP1, HDAC9, FOXO1, ARNTL, FOXS1, RASD1, DNAJB6, DEAF1, ATOH8, SUDS3, SCAI, RUNX1T1, TCF7L2, HSPA8, ZBTB14, ZBTB16, NR1D2, TRERF1, HEYL, PER1, BCL6, ID2, ZNF219, ID4, UBA3, NOSTRIN, ZNF217, MDM4, MPHOSPH8, EIF4G2 |
| GO:0072540 | T-helper 17 cell lineage commitment | 0.01265 | IL6, STAT3, SLAMF6, IL6R |
| GO:0009755 | hormone-mediated signaling pathway | 0.01323 | GHR, CRHBP, LHCGR, NR5A2, THRA, GCGR, FSHB, RARB, NR1D2, PPARD |
| GO:0071526 | semaphorin-plexin signaling pathway | 0.01358 | SEMA6B, FARP2, SEMA4D, SEMA4B, SEMA6D, SEMA4C, SEMA3E, MET |
| GO:0050832 | defense response to fungus | 0.01358 | ZBP1, DEFA6, TSLP, COTL1, S100A12, IL17RC, S100A9, S100A8 |
| GO:0051973 | positive regulation of telomerase activity | 0.01358 | GREM1, CCT2, HSP90AA1, MYC, PTGES3, TCP1, PRKCQ, CCT4 |
| GO:0051085 | chaperone mediated protein folding requiring cofactor | 0.01358 | HSPA8, DNAJB1, HSPH1, HSPA1L, HSPA5, DNAJB5, PTGES3, HSPE1 |
| GO:0007507 | heart development | 0.01409 | POPDC3, SPARC, TRAF3IP2, HSPB7, ADM, CACNA1C, HDAC9, PDLIM1, ERBB3, ERBB2, DVL1, SMYD2, TNNI3, SOX9, MAP2K3, MKKS, EDN1, CXADR, OSR1, CACYBP, SOD2, ID2, MYL2, HAND1, ATM, EIF4G2, PPARD |
| GO:0035928 | rRNA import into mitochondrion | 0.01691 | PNPT1, TST, MRPL18 |
| GO:0007339 | binding of sperm to zona pellucida | 0.01793 | ACR, ADAM2, CCT2, HSPA1L, TCP1, ZP3, CCT5, ZPBP, CCT4 |
| GO:1990573 | potassium ion import across plasma membrane | 0.01793 | KCNK5, KCNH2, SLC12A2, ATP4A, KCNJ10, KCNJ15, KCNJ16, SLC12A6, KCNJ2 |
| GO:0034620 | cellular response to unfolded protein | 0.01798 | ERN1, HSPA8, HSPA1L, HSPA5, BAG3, HSF1, TM7SF3 |
| GO:0014912 | negative regulation of smooth muscle cell migration | 0.01884 | IGFBP5, SEMA6D, IGFBP3, SLIT2, PPARD |
| GO:0061684 | chaperone-mediated autophagy | 0.01908 | HSPA8, HSP90AA1, BAG3, LAMP2 |
| GO:0044321 | response to leptin | 0.01908 | EDN1, CCND1, STAT3, LRP2 |
| GO:0039536 | negative regulation of RIG-I signaling pathway | 0.01908 | NLRX1, RNF125, SEC14L1, DHX58 |
| GO:0030522 | intracellular receptor signaling pathway | 0.01918 | MYLIP, STAT3, ESRRB, NR1D2, AHR, DCBLD2, NR4A2, NR4A1, NR5A2, TBL1XR1, SCD, PLIN1, PPARD |
| GO:0006509 | membrane protein ectodomain proteolysis | 0.01944 | P2RX7, ADAM17, ERAP1, ADAM10, PRKCQ, PSEN1 |
| GO:0036092 | phosphatidylinositol-3-phosphate biosynthetic process | 0.01944 | FIG4, UVRAG, PIK3R4, ATM, PIK3CB, PIK3C2B |
| GO:0045444 | fat cell differentiation | 0.01950 | NR4A2, BBS2, TCF7L2, NR4A1, MKKS, CCND1, CEBPD, ERAP1, LRRC8C, ID4, OSBPL11, FOXO1 |
| GO:0042531 | positive regulation of tyrosine phosphorylation of STAT protein | 0.01950 | GH2, GHR, SOCS3, IL6, HDAC2, TSLP, HSF1, STAT3, HCLS1, IGF1, IL6R, TNFRSF1A |
| GO:0002931 | response to ischemia | 0.01950 | P2RX7, MAP2K3, CASP9, GPR151, TIGAR, EEF2K, P2RX2, EIF4EBP1, AKT1, MLYCD, PIK3CB |
| GO:0032722 | positive regulation of chemokine production | 0.02038 | IL1RL1, APP, IL6, ADAM17, IL4R, TSLP, IL6R, HIF1A, MYD88 |
| GO:0006412 | translation | 0.02050 | HBS1L, MRPL18, RPLP1, RPL12, RPLP0, RPL10A, RPL7, RPL7A, RPS17, RPS19, AKT1, RPL35, EIF4EBP2, IGF2BP3, RPS2, RPS27A, RPS5, RPS6, DHPS, RPL23A, RPS3A, MRPS5, MRPL52, RPL24, RPS20, RPL29, CPEB2 |
| GO:0006006 | glucose metabolic process | 0.02163 | PDHA1, OAS1, MYC, DCXR, KBTBD2, PDK4, AKT1, OMA1, PDHB, BRS3, PPARD |
| GO:0050804 | modulation of synaptic transmission | 0.02163 | SRGN, NLGN3, PTPRD, PXK, NLGN1, RELN, GRIK5, PACSIN2, EIF4EBP2, GRIK1, PPP1R9A |
| GO:0051131 | chaperone-mediated protein complex assembly | 0.02360 | CCT2, MKKS, HSP90AA1, BBS10, HSPA4, PTGES3 |
| GO:1900029 | positive regulation of ruffle assembly | 0.02415 | P2RY12, NLGN1, COBL, FAM98A, EPS8L3 |
| GO:0045806 | negative regulation of endocytosis | 0.02415 | LGALS3, MTMR2, PACSIN2, PRKD1, EPHA3 |
| GO:0048870 | cell motility | 0.02438 | ADAM17, ITGB4, CFL2, ENPP2, FSCN1, ELMO1, PLD1, PLD2 |
| GO:0006479 | protein methylation | 0.02486 | PCMTD2, METTL22, PRMT2, FAM98A, SNRPB, CAMKMT, PCMTD1 |
| GO:0009410 | response to xenobiotic stimulus | 0.02675 | HDAC2, TRAF3IP2, TNFRSF11B, AHR, ACACB, PNP, CCND1, CDH1, MYC, GNA12, TIMP4, BCHE, TIGAR, EMX2, ITGA2, MTHFR, SOD2, DUSP6, P2RX7, GNPAT, ADAM17, CREB1, NPC1, RPS6KB1, SST, PDE3A, TXNIP, PLIN2 |
| GO:0071314 | cellular response to cocaine | 0.02701 | CCNA2, CRHBP, PPP1R1B, TGM2 |
| GO:0034214 | protein hexamerization | 0.02701 | UGDH, LRRC8C, LRRC8D, LRRC8A |
| GO:0032287 | peripheral nervous system myelin maintenance | 0.02701 | FA2H, SH3TC2, AKT1, PLEC |
| GO:0060296 | regulation of cilium beat frequency involved in ciliary motility | 0.02701 | BBS2, CATSPER1, MKKS, GAS2L2 |
| GO:0060075 | regulation of resting membrane potential | 0.02701 | KCNK5, KCNJ10, PSEN1, KCNJ2 |
| GO:0016601 | Rac protein signal transduction | 0.02829 | FARP2, HACE1, ALS2, ELMO1, RAC2, WASF2 |
| GO:0007417 | central nervous system development | 0.02851 | SEMA6B, APP, EMX2, MOG, CNTN6, ZBTB16, SLC2A1, ATN1, LIG4, GRIK1, CAPG, ZIC5, GBA2, HAPLN1, KLK6, UGT8, RELN, TIMP4, SH3GL1 |
| GO:0009615 | response to virus | 0.02888 | IVNS1ABP, IFITM3, URI1, IFITM2, IFNB1, IRAK3, IFIT1, IFIT2, IFIH1, OAS1, PRKRA, HYAL2, DHX58, ITGB8, CCT5, MYD88 |
| GO:0006909 | phagocytosis | 0.02903 | CDC42SE1, KIAA1109, CD93, ANXA3, SLC11A1, CEACAM4, ELMO1, PECAM1, MET, MYD88, CDC42SE2 |
| GO:2000737 | negative regulation of stem cell differentiation | 0.03028 | LBH, STAT3, ESRRB, HNRNPU, NELFB |
| GO:0051146 | striated muscle cell differentiation | 0.03028 | SPAG9, RB1, POPDC3, IGFBP5, AKT1 |
| GO:0009267 | cellular response to starvation | 0.03080 | HSPA8, RRAGC, GCGR, VPS41, LAMP2, CLEC16A, PDK4, NUPR2, FNIP1, DNAJC15, FOXO1, PIK3C2B |
| GO:0002576 | platelet degranulation | 0.03089 | SRGN, SERPINA3, APP, SPARC, VWF, TGFB3, IGF1, SYTL4, TTN, TF, CD109, LAMP2, PECAM1, SERPING1, TIMP3, TAGLN2, TIMP1 |
| GO:2001016 | positive regulation of skeletal muscle cell differentiation | 0.03210 | MIR1-1, GPC1, ARNTL |
| GO:0035585 | calcium-mediated signaling using extracellular calcium source | 0.03210 | P2RX7, P2RY12, CACNA1C |
| GO:0000915 | actomyosin contractile ring assembly | 0.03210 | ANLN, PDCD6IP, RACGAP1 |
| GO:0050731 | positive regulation of peptidyl-tyrosine phosphorylation | 0.03268 | CSF3, SEMA4D, CD81, IGF1, ICAM1, GHR, IL6, RELN, ENPP2, PECAM1, HCLS1, CSPG4, ARHGEF2, IL6R |
| GO:0032868 | response to insulin | 0.03352 | FABP3, STXBP3, INSIG2, VPS13C, GCNT1, KBTBD2, SLC2A1, TNFSF10, ADM, SRSF6, KLF15, ICAM1 |
| GO:0044772 | mitotic cell cycle phase transition | 0.03354 | CCNA2, CCNA1, CCND3, CCND1, CCNE2, CKS1B |
| GO:0070542 | response to fatty acid | 0.03354 | GNPAT, FABP3, INSIG2, SCD, SOX9, FOXO1 |
| GO:0006893 | Golgi to plasma membrane transport | 0.03354 | RABEP1, RAB3IP, GOPC, GOLGA7, EXOC2, EXOC1 |
| GO:1904851 | positive regulation of establishment of protein localization to telomere | 0.03641 | CCT2, TCP1, CCT5, CCT4 |
| GO:0015807 | L-amino acid transport | 0.03641 | SLC43A2, SLC38A9, SLC7A1, SLC7A2 |
| GO:0000301 | retrograde transport, vesicle recycling within Golgi | 0.03641 | COG7, COG6, COG4, COG3 |
| GO:0030502 | negative regulation of bone mineralization | 0.03725 | SRGN, GREM1, SOX9, LTBP3, HIF1A |
| GO:0007528 | neuromuscular junction development | 0.03814 | PPFIBP2, ALS2, P2RX2, ERBB2, DVL1, AGRN, SNTA1 |
| GO:0042127 | regulation of cell proliferation | 0.03950 | STAT5A, IL4R, TGFB3, FANCL, STAT3, HP1BP3, OSGIN1, GRPR, FA2H, CCDC88A, SQLE, CCND3, NUAK1, NR5A2, ERBB3, BCL6, TXNIP, SOX9, ARHGEF2, BIRC2 |
| GO:0032728 | positive regulation of interferon-beta production | 0.03997 | IFIH1, HSP90AA1, POLR3B, OAS1, POLR3D, DHX58, HMGB2, POLR3G |
| GO:0051726 | regulation of cell cycle | 0.04026 | RB1, INO80C, BIRC8, ARNTL, ING4, MNT, ING3, SKP2, CLIC1, CDK18, HSPA8, PNPT1, GADD45A, PLK1, STAT3, CABLES1, DHCR24, GADD45G, ZZZ3, CCNE2, MORF4L2, UBA3, HBP1, ATM, MDM4, NUPR2, DTL, BIRC2, EIF4G2 |
| GO:0001666 | response to hypoxia | 0.04137 | ADSL, TGFB3, ITGA2, SLC2A1, MTHFR, ADM, HIF3A, SOD2, HIF1A, CYGB, HSP90B1, NR4A2, PDLIM1, TGFBR3, DPP4, ADAM17, CREB1, P2RX2, DDIT4, ATM, ANGPTL4, BIRC2 |
| GO:0051965 | positive regulation of synapse assembly | 0.04319 | NLGN3, PTPRD, NLGN1, EEF2K, FLRT2, STAU2, SLITRK1, IL1RAPL1, SYNDIG1, THBS2 |
| GO:0035924 | cellular response to vascular endothelial growth factor stimulus | 0.04340 | ERN1, MAP2K3, NR4A1, SPRY2, AKT1, MT1G, PRKD1 |
| GO:0019933 | cAMP-mediated signaling | 0.04340 | PDE3A, EIF4EBP2, ADM, AHR, SOX9, PDE7A, TGM2 |
| GO:0000079 | regulation of cyclin-dependent protein serine/threonine kinase activity | 0.04439 | CCNA2, CCNA1, CCND3, BLM, CCNK, CCND1, CCNE2, CCNH, GADD45A |
| GO:1901998 | toxin transport | 0.04469 | DNAJA1, CCT2, HSPA5, ANTXRL, TCP1, ADAM10, CCT5, CCT4 |
| GO:0051895 | negative regulation of focal adhesion assembly | 0.04506 | BCAS3, DLC1, FAM107A, ARHGAP6, CLASP2 |
| GO:0001678 | cellular glucose homeostasis | 0.04573 | STXBP3, LRRC8D, LRRC8A, HIF1A, KLF15, FOXO1 |
| GO:0006027 | glycosaminoglycan catabolic process | 0.04573 | IDUA, GPC1, HYAL2, SDC2, AGRN, GPC6 |
| GO:0031023 | microtubule organizing center organization | 0.04726 | BCAS3, MPDZ, CLASP2, CDK5RAP2 |
| GO:0015810 | aspartate transport | 0.04726 | LRRC8C, LRRC8D, LRRC8A, SLC25A13 |
| GO:0097105 | presynaptic membrane assembly | 0.04726 | NLGN3, PTPRD, NLGN1, IL1RAPL1 |
| GO:1904871 | positive regulation of protein localization to Cajal body | 0.04726 | CCT2, TCP1, CCT5, CCT4 |
| GO:1901381 | positive regulation of potassium ion transmembrane transport | 0.04910 | KCNH2, KCNE1, KCNJ10, KCNJ15, KCNJ16, KCNN3, KCNJ2 |

| GO ID | Term | P-value | Genes |
| --- | --- | --- | --- |
| CC |  |  |  |
| GO:0005829 | cytosol | 0.00000 | RB1, CDA, IPO11, ZFYVE9, HNRNPU, LIPA, SMC2, RPL7, DPYSL5, ZMIZ1, GPBP1, AKT1, RPL35, MLYCD, CEP97, VPS36, RNF111, CDK5RAP2, STARD4, WSB1, DICER1, GTPBP1, UPB1, ZFYVE16, MYL2, PARD3, DDIT4, MYL3, RPL24, TXNIP, NHLRC1, PRKCQ, PRKD1, RPL29, CLOCK, GNE, ZNF397, URI1, MTMR2, BCL10, ADH7, AAAS, HIF1A, SULT1A1, PPP4C, HSPH1, AFMID, S100A12, ZNF146, MCTS1, STARD13, CHKA, HOMER1, PLK1, RAB3IP, MTHFR, TYSND1, DHFR, PPFIBP2, GJB6, PAH, PLCH1, STRN, MTMR10, PIGN, RPLP1, RPLP0, PYGM, PYGL, HIF3A, OSBPL11, PHF7, ADO, ARHGAP6, WDR44, IFIH1, ING4, IMPA2, TNKS1BP1, EMD, SULT1C4, APC2, MIOS, ANXA2, HSBP1, COPZ2, EPS15L1, SERPINB8, DUSP6, ITPKC, AGPS, TCP1, HCLS1, PLIN2, PMP2, PLIN1, EXOC2, EXOC1, AHNAK, SAMD9, CEP295NL, NIP7, RPL12, PAPSS2, EXTL2, SNX27, CARNS1, MICALL2, RPS2, RPS27A, FARP2, TIGAR, HSPA1L, KIDINS220, TRAPPC10, STAT3, SAMD4B, ELP2, FMO5, BYSL, TTLL7, FABP3, SNX16, CENPL, C16ORF89, CENPN, PKN1, CRLF3, PLCD4, DGKG, ANKRD37, ALDH1L1, CSE1L, GMNN, LTN1, HIKESHI, AHR, RPL10A, GRB14, PAPOLG, PIM1, PLS3, ZNF687, MAF1, NUDT14, MAP2K3, MYLIP, RPS5, HARBI1, RPS6, FBXO17, AKR1E2, OLFM4, MYT1, S100A6, FSCN1, ANAPC5, FKBP4, S100A9, S100A8, SNRPB, SHC2, BLM, CUL5, CUL1, RHOBTB3, STK39, GMPR, ACAT2, FOXO1, HSP90B1, STIP1, SOCS3, BAG2, BAG3, STK36, PMVK, DVL1, SMYD2, EIF4EBP1, EVI5, ANKRD28, CD163, TIA1, ZBTB14, OSBPL3, ZBTB16, ESRRB, NRDC, HPS4, DHCR24, COPS3, LSM7, RGCC, CNOT2, TADA1, TUBGCP4, XAF1, EIF4G3, MYD88, EIF4G2, RNF13, SH2D4A, CALCOCO2, AMD1, PIK3CB, DUSP16, PIK3C2B, RPL7A, DUSP10, AP1G1, PSTPIP2, PRKG2, UNKL, CEP290, SPG11, ADSL, HSP90AA1, PDCD6IP, HMGCS1, GGPS1, CKAP2L, VPS13C, DHPS, AHSA1, MTFR1, PPP1R12B, DTL, BIRC2, CARNMT1, PBXIP1, NSL1, TTN, HDAC7, DNAJB1, FCHO1, DNAJB6, DNAJB5, IGF2BP3, TNNI3, POLR2H, IGF2BP2, SLC25A20, FNIP1, WASF2, SLC12A2, SPAG9, PPP1R14A, FEM1A, SMOX, ERAP1, CABLES1, RPL23A, AKR7L, CPPED1, DNAJA1, MYO1D, POLR3B, IMPDH1, POLR3D, VPS41, KLF9, RAB13, POLR3G, METAP1, GLTP, APP, MTRR, ECI2, PREP, DIXDC1, PPP2R2A, ZDHHC8, GCC1, IFIT1, GBA2, IFIT2, HERC4, RPS17, MT2A, CCDC91, RASSF2, PNP, FRAT2, RPS19, HERC1, FTH1, DPF2, SKP2, ATP6V1E2, TBC1D10A, HERC6, COG4, NFKBIL1, COG3, YOD1, KNL1, GNPAT, MT1A, RRAGC, CD109, TAGLN2, TRIB3, FLYWCH1, TRIM11, EPHA3, TMEM87A, NECAB1, GYPA, SLC22A5, STXBP3, MAOA, PIK3R4, TWF1, FXR1, SEC14L1, RACGAP1, DPH3, SNX5, BBS2, SRPK2, STAT5A, MICAL3, SMARCA5, NBEAL1, STAM, TRERF1, SRPK1, ANLN, POLA1, CCDC88A, PEX5L, CDAN1, AKR1B10, ID2, PPP1R1B, PDE3A, RPS20, ATM, INTS9, FBXL4, HBS1L, ACY3, C2CD5, HIP1, UBXN1, C2CD3, SLC44A1, HILPDA, HBB, KRT28, HYAL2, NMRK1, RAC2, KIF13B, RAB8A, CCT2, PRMT2, BHMT2, TSC22D3, TFE3, NME3, CACYBP, RHOC, RENBP, APRT, DUS2, UGDH, RAD51C, POC5, GAN, G2E3, ARHGEF2, DYNC1I2, ADH1B, INPP1, HSD17B4, NPL, C10ORF90, EXOSC6, GPC1, HSF1, LMNA, METTL4, CCT5, CCT4, RANBP2, MON2, MAP3K1, MPP4, GCA, PLEKHA1, PTGES3, UBE2G1, SORBS3, ALDH4A1, PER1, ITCH, CAPZA3, KLHL5, PRKRA, DLC1, MPHOSPH8, PNPLA2, OTUD4, CRTC1, NRL, ARID4B, COMT, NR3C1, CNDP1, CCND1, COTL1, DENND5A, PDE8A, SH3GL1, TGM2, ZBP1, OSR1, KCTD5, MID1, PDIA6, KRT73, KCTD7, CAMKMT, ADAM17, CCNE2, DOK5, CARD19, ELMO1, STAM2, PPID, IDI1, COPA, PXK, AKNA, UBA6, PXN, CLEC16A, DAPP1, DTX4, PDS5A, ACACB, ACACA, NCKIPSD, DNAJC21, PACSIN2, SUDS3, IVNS1ABP, HSPA8, SMAD1, STIL, PNPT1, HSPA5, HSPA4, ALYREF, AZIN2, DNAJC13, PAN2, QDPR, NR4A1, ARHGAP31, DLG1, GNB2, UBA3, PICALM, GPSM2, USP37, THRA, USP32, NGLY1, SLC2A1, PPP1R9A, CHTF18, YBX3, GHR, CASP9, SPRED1, EEF2K, PPP2R5E, CA2, BLNK, KLF11, TTC37, MKKS, USP48, EED, RBM18, ARHGEF17, PLA2G4C, ARAP3, RPS3A, GPCPD1, CCNA2, CCNA1, ALS2, OAS1, MYH8, FBP1, FAN1, PLEC, KRTAP21-2, HSP90AB4P, RAP1GDS1, ASAP3, RBM4B, CYGB, USP19, IL1RL1, TAF1D, MTA1, DNPEP, ERBB2, BUB3, MAPK4, GBP4, CLASP2, ARFGEF1, SPRY4, SCLT1, USP28, FMNL2, RPS6KB1, SPRY2, NAA15, PDE7A |
| GO:0016020 | membrane | 0.00000 | GLTP, CNTFR, APP, SPARC, LPGAT1, ECI2, PREP, HNRNPU, ICAM1, RPL7, LGALS3, RPS17, KIAA1109, CCDC91, RPS19, HERC1, ARL6IP5, PIEZO2, PIEZO1, RPL35, DIP2A, DIP2B, AGFG2, DDX18, AQR, ENTPD4, IL1R1, COG3, ACSL5, GTPBP1, SYTL4, GNPAT, RPL24, PRKD1, RPL29, TMEM87A, MTMR2, GYPA, PIK3R4, OMA1, PTTG1IP, AAAS, HSD11B1, FXR1, CYB5R2, CYB5R3, NEU1, SYNDIG1, BBS2, PPP1R21, NBEAL1, TYSND1, HNRNPM, FA2H, CCDC88A, RPS20, STRN, SLC44A5, HBS1L, PYGB, MTMR10, SLC44A3, HIP1, SLC44A1, PIGN, SLC44A2, RPLP0, PTH1R, VLDLR, OSBPL11, PEAR1, PCSK6, GLIPR1, RAC2, CLIC1, EMD, PCMTD1, ELOVL1, ANXA2, ANXA3, KREMEN1, NIPA1, EPS15L1, CEACAM4, AGPS, EXOC2, EXOC1, POPDC3, AHNAK, SLC20A2, STAU2, PRIM1, RPL12, ATL2, HEATR1, AKAP8, HSD17B4, SLC7A1, ERMP1, SNX27, MGAT5, CXCR2, CD59, RPS2, METTL7A, RPS27A, RANBP2, BCHE, SLC16A1, PLEKHA1, KIDINS220, PKDREJ, MLH1, BYSL, FIG4, SLC8A3, ITCH, MPHOSPH9, RIT2, CAPZA3, CR1L, PRKRA, DLC1, HNRNPA2B1, PNPLA3, C16ORF89, GOPC, PLP2, PNPLA2, DGKG, RERGL, ITPRIP, ATP8A1, CD81, CSE1L, MSMO1, RPL10A, COMT, FADS3, CCND3, CCND1, DMBT1, CDH1, PAPOLG, LAMP2, ENPP4, SLC39A1, SLC16A3, IL6R, MAP2K3, UNC5CL, RPS5, RPS6, ADAM10, TOM1L2, ADAM17, MSH2, DNAJB11, SLCO2A1, ELMO1, CLEC4E, SLC27A3, TTPAL, RASL12, COPA, SLC26A2, AKNA, CNP, PSEN1, PLD1, CYP19A1, HSP90B1, DPP4, MAN2A1, BAG3, PMVK, DRD4, MARK2, HSPA8, CD163, MGAM, HSPA5, OSBPL3, ALYREF, HPS4, EPHX3, DHCR24, DNAJC13, DLG1, IBTK, GNB2, CNOT2, SLCO1A2, CSPG5, TUBGCP4, CLPTM1L, STT3B, LRP10, SERINC2, TMEM19, EIF4G2, PICALM, CALCOCO2, USP32, SLC2A1, PIK3CB, CMAHP, SLC2A5, CHTF18, PIK3C2B, RPL7A, MFSD6, AP1G1, PSTPIP2, BLNK, CEP290, SNTA1, HSP90AA1, PDCD6IP, ITGA2, ABCC5, PLA2G4C, COBL, TNFRSF1A, DNM3, DDX19B, NPC1, OAS1, ALS2, TMBIM1, MAN1B1, MET, PLEC, MOSPD2, ATP10B, PBXIP1, LRP2, NCLN, GNG5, DNAJB6, CHSY1, LRRC8C, LRRC8D, STOM, LRRC8A, SLIT2, CLASP2, GALNT7, SLC12A2, KCNJ10, SEMA4B, ERAP1, SPRY4, MBOAT1, KCNJ15, KCNJ16, HSPE1, DLK1, DNAJA1, P2RX7, CLCN4, ABHD17B, SCD, VPS41, SPRY2, NAA15 |
| GO:0005737 | cytoplasm | 0.00000 | IPO13, IPO11, TESK2, RBPJ, PTPDC1, SMC2, RPL7, DPYSL5, ZMIZ1, WDR90, AKT1, RTTN, DIP2B, MLYCD, RNF111, CDK5RAP2, MT1HL1, STARD4, CXADR, STARD9, IFT80, DICER1, EML2, EML3, FEZ1, MYL2, DDIT4, RPL24, TXNIP, PRKCQ, PRKD1, CLOCK, GNE, ZNF397, CDC42SE1, URI1, MTMR2, FPR1, PHYHIPL, SLAIN1, BCL10, ADH7, HIF1A, SULT1A1, PRPSAP2, CYP2B6, HSPH1, CYB5R3, AFMID, S100A12, CDK18, PLK3, STARD13, HACE1, CHKA, HOMER1, CYP4F2, PLK1, NR1D2, A1CF, SETX, HNRNPM, GJB1, TDP1, NAPEPLD, NOSTRIN, PLCH1, CPEB2, CDC42SE2, RNF220, PYGB, GMEB1, MTMR10, RPLP0, WDR41, ADM, PYGM, PTH1R, PYGL, ARHGAP6, TRIOBP, TAF7L, IFIH1, PCMTD2, TUBA1B, NHSL2, IMPA2, TRIM2, MMP28, TNKS1BP1, NELFB, EMD, PCMTD1, SULT1C4, APC2, MIOS, ANXA2, ANXA3, ELMOD3, EPS15L1, SERPINB8, DUSP6, TEX19, ITPKC, RARB, HCLS1, PMP2, EXOC1, AHNAK, SAMD9, PRUNE2, PAIP2, RPS27A, RNF130, DTD2, FARP2, TIGAR, HSPA1L, STAT3, SAMD4B, ELP2, PLXDC1, BYSL, HEYL, RNF148, GOPC, PKN1, CRLF3, PLCD4, PLCD1, ALDH1L1, CSE1L, GMNN, ATN1, FAM107A, AHR, CDC14C, MT1DP, GRB14, CDH1, CFL2, PIM1, PAPOLA, PLS3, HMGN2, MAF1, HMGN1, SEMA6D, USP3, FBXO17, THOC1, VASH2, VASH1, FRMD4B, MOB3B, FRMD6, LIX1, S100A6, FSCN1, SRSF3, ADAM8, FKBP4, S100A9, SRSF7, SNRPB, BLM, CNP, BIRC8, ACAT2, FOXO1, KLC4, BAG3, STK36, SMYD2, EIF4EBP1, EIF4EBP2, SLC38A2, MPDZ, UVRAG, TIA1, FANCL, CCDC14, ESRRB, HPS4, PXDNL, DHCR24, COPS3, IBTK, RGCC, CNOT2, ZFP64, ANXA2P2, TUBGCP4, MYD88, EIF4G2, PIWIL2, SH2D4A, CALCOCO2, AKAP8L, PIK3CB, DUSP16, PIK3C2B, RPL7A, LBH, DUSP10, SGCA, AP1G1, PSTPIP2, EMILIN3, CEP290, SPG11, BCAS3, HSP90AA1, HMGCS1, VPS13C, LANCL1, DHPS, PATL2, LRTOMT, ZNF638, HAND1, IL1RAPL1, NUMB, PPP1R12B, PPP1R12C, BIRC2, TRIM55, CEBPB, HDAC2, PPM1H, HDAC9, HDAC7, DNAJB1, FCHO1, DNAJB6, LRRC8C, LRRC8D, IGF2BP3, IGF2BP2, FNIP1, SPAG9, PPP1R14A, MMAA, SMOX, NCKAP5L, ERAP1, RPL23A, AKR7L, TPD52L1, MYO1D, P2RX7, MYO1B, IMPDH1, NOVA1, NAGS, METAP1, GLTP, APP, SPARC, LPGAT1, TP53RK, CCDC89, MT1M, PREP, MT1X, IFIT1, IFIT2, LGALS3, MT2A, RASSF2, PNP, FRAT2, RPS19, HERC1, FTH1, RASSF5, CHEK2, DHX58, TRIM23, LIMCH1, ARRDC3, YOD1, JMJD8, MT1A, RRAGC, MT1G, DNA2, MT1B, TRIM11, MT1E, NECAB1, SLC22A5, HEPACAM, TWF1, LPAR2, PTTG1IP, KLK6, FXR1, NUAK1, SEC14L1, SEC14L4, NEU1, DPH3, KCNN3, SCAI, BBS2, SRPK2, STAT5A, DTNA, GADD45A, SRPK1, GADD45G, CCDC88A, CDAN1, ID2, PPP1R1B, ID4, INTS7, ATM, RAB40AL, ACY3, HIP1, UBXN1, CCDC125, KIF19, HYAL2, NMRK1, RAC2, KIF13B, CLIC1, EDN1, PRMT2, FSHB, ZFR, TFE3, CACYBP, MTSS1, APRT, DUS2, NR5A2, RAD51C, POC5, GAN, ARHGEF2, KIZ, DYNC1I2, C10ORF88, NOXO1, PRKX, CAPG, GNAI1, ARNTL, C10ORF90, RELN, HSF1, MAP4K5, CCT5, RANBP2, UBL4B, MAP3K1, GCA, PLEKHA1, MTX2, RFTN1, TUBE1, KLHL4, ACTL8, SORBS3, PER1, ITCH, RIT2, KLHL5, CR1L, PRKRA, DLC1, HNRNPA2B1, PNPLA3, MPHOSPH8, PNPLA2, NDUFA13, CRTC1, CIB2, NRL, NR3C1, RGS5, CCND3, CCND1, DMBT1, SH3GL1, ZBP1, UNC5CL, SLC30A4, ADAM10, KCTD5, MID1, ERMN, KCTD7, CAMKMT, ERN1, ADAM17, CCNE2, DOK5, ELMO1, STAM2, CARS2, PPID, IDI1, COPA, PXK, UBA6, PXN, CACNA1C, DEAF1, DNAJC21, ISOC1, PACSIN2, GAS2L2, CD99, TBC1D15, MARK2, HSPA8, SMAD1, STIL, PNPT1, HSPA5, HSPA4, ALYREF, SLC52A3, AZIN2, TDRD7, KLHL24, CLK1, QDPR, NR4A2, NR4A1, TBCK, DLG1, GNB2, UBA3, MAP6D1, GPSM2, NGLY1, HSPB7, HMGB2, HMGB3, CMAHP, PPP1R9A, YBX3, TTC28, RBM3, CASP9, HEBP2, C20ORF203, EEF2K, PPP2R5E, CA2, BLNK, SNTA1, TTC37, MKKS, ARAP3, RPS3A, IRAK3, GPCPD1, DNM3, CCNA2, TGFBR3, CCNA1, C12ORF4, DDX19B, OAS1, MYH8, ATG4C, NCBP3, FBP1, PLEC, HSP90AA2P, ASAP3, ASAP2, CYGB, PDLIM1, RMND5A, MTA1, DNPEP, MCRIP2, GNA12, MKNK2, ATOH8, RGS20, SLIT2, EPS8L3, MAPK4, CLASP2, LIMK2, SPRY4, DLK1, RPS6KB1, QRICH2, NAA15, PMFBP1 |
| GO:0005654 | nucleoplasm | 0.00003 | RB1, SMARCAL1, CCNK, IPO11, MTRR, TP53RK, CCNH, HNRNPU, TESK2, PPP2R2A, RBPJ, LIPA, ELK1, PTPDC1, SMC2, RPS17, CCDC91, RASSF2, RPS19, ZMIZ1, MYC, ZMIZ2, CHEK2, DPF2, AKT1, SOX9, CCNL2, SKP2, RNF111, SCN1A, HERC6, AQR, CXADR, NFKBIL1, SLC35C2, ACSL5, KNL1, RRAGC, TRIB3, DNA2, FLYWCH1, AURKAIP1, CLOCK, TRIM11, EPHA3, NECAB1, URI1, GYPA, SYCP2L, ZBTB1, ZNF22, PTTG1IP, AAAS, HIF1A, NUAK1, HSPH1, SEC14L1, PPP4C, RACGAP1, DPH3, HOXC9, SCAI, NKX2-2, SRPK2, STAT5A, PLK3, DTNA, GADD45A, PLK1, MICAL3, SMARCA5, NR1D2, TRERF1, A1CF, SRPK1, SETX, HNRNPM, ANLN, POLA1, ZZZ3, ID2, TDP1, NAPEPLD, ID4, INTS4, RPS20, INTS7, HBP1, ATM, NOL11, INTS9, ATG2B, ZNF131, GMEB1, UBXN1, SLC44A1, GRIK5, HILPDA, HIF3A, CTCF, OSBPL11, PHF7, ING4, ING3, NELFB, EMD, TEAD3, WDR36, PBRM1, ZHX3, MIOS, PRMT2, HSBP1, TFE3, CACYBP, MED29, DUSP6, APRT, RAD51B, MRPL52, UGDH, MED25, RAD51C, NR5A2, POC5, MED20, RARB, HNRNPH3, RAPGEF5, PPARD, WDR27, PLPPR1, ADH1B, SATB1, NIP7, PRIM1, HEATR1, AKAP8, WDR20, CAPG, PRKX, ARNTL, C10ORF90, EXOSC6, EXTL2, MNT, SNX27, GPC1, HSF1, LMNA, CXCR2, PCGF1, RPS2, RPS27A, SF3B1, CCT4, RUNX1T1, RANBP2, UTP6, HSPA1L, PLEKHA1, NFYB, PTGES3, STAT3, ELP2, MLH1, BYSL, PER1, HEYL, ITCH, PRKRA, ZNF219, HNRNPA2B1, KLHL8, CENPL, ZNF217, TRIP11, PRPF31, CENPN, PKN1, MPHOSPH8, ZNF354C, PNPLA2, ANKRD37, NDUFA13, CRTC1, DSCC1, POGK, CSE1L, GMNN, ATN1, NRL, ARID4B, HIKESHI, AHR, NR3C1, CCND3, CCND1, PAPOLG, PAPOLA, PIM1, MACROD1, SLC16A7, OIP5, ZNF687, MAF1, HMGN1, TLE4, MAP2K3, IL4R, ZBTB38, RPS5, USP3, RPS6, THOC1, LIG4, THOC2, OLFM4, MYT1, CAMKMT, MSH2, CCNE2, TBL1XR1, MORF4L2, SNRNP27, SRSF3, ANAPC5, FKBP4, SRSF6, STAM2, S100A9, SRSF7, PPID, ABCG2, SNRPB, INO80C, BLM, DNMT1, AKNA, STK39, CUL1, PSEN1, DTX4, PDS5A, FOXO1, SEL1L3, DEAF1, SMYD2, SUDS3, MARK2, BCL9L, ANKRD28, IVNS1ABP, HSPA8, SMAD1, TIA1, ZBTB14, FANCL, ALYREF, ESRRB, BTBD8, PHOX2B, NR4A2, NR4A1, COPS3, IBTK, LSM7, RGCC, BCL6, CNOT2, UVSSA, TADA1, MDM4, CSPG4, XAF1, MXD4, ACADVL, USP37, RNF13, THRA, TSEN15, HSPB7, HMGB2, C1ORF174, PIK3CB, CHTF18, DUSP16, CKS1B, PIK3C2B, RBM3, SPRED1, DUSP10, POLL, BCAS2, KLF11, TTC37, USP48, HSP90AA1, EED, RBM18, PLA2G4C, RPS3A, KLF15, ETV5, CCNA2, CCNA1, METTL22, DDX19B, CREB1, OAS1, ZNF638, HAND1, POLR1E, LCOR, PPP1R12B, DTL, FAN1, CEBPB, HDAC2, CEBPD, PPM1H, ASAP3, RBM4B, PDHB, HDAC9, HDAC7, DNAJB1, TAF1D, RMND5A, MTA1, FCHO1, DNAJB6, TRA2B, TRA2A, TAF1A, MKNK2, ATOH8, POLR2H, BUB3, MAPK4, ARFGEF1, TCF7L2, KDM4C, KDM4D, SMOX, TAF13, LYSMD1, USP28, U2SURP, RAD52, RPS6KB1, POLR3B, NOVA1, POLR3D, KLF9, POLR3G |
| GO:0022626 | cytosolic ribosome | 0.00006 | RPLP1, RPS5, RPL12, RPS6, RPLP0, RPL23A, RPS3A, RPL10A, RPL7, RPL7A, RPS17, RPS19, RPL24, RPL35, RPS20, RPS2, RPL29, RPS27A |
| GO:0005856 | cytoskeleton | 0.00010 | DGKG, HIP1, CALCOCO2, KCNC3, DIXDC1, CMAHP, PPP1R9A, SGCE, SGCA, KRT28, PSTPIP2, KIF13B, ARL6IP5, RAC2, CEP290, CDK5RAP2, SNTA1, MYLIP, HSBP1, VASH2, ARAP3, ELMOD3, FRMD4B, RHOC, EML2, ERMN, EML3, FRMD5, FRMD6, GAN, MYL2, PARD3, FSCN1, TAGLN2, ARHGEF2, PPP1R12B, S100A9, PPP1R12C, S100A8, PLEC, CDC42SE1, PXN, RHOBTB3, STK39, TWF1, SLAIN1, PDLIM1, CNN1, SNX27, FCHO1, PACSIN2, S100A12, STOM, IGF2BP2, PLEKHH1, WASF2, MPZL2, IVNS1ABP, BBS2, FARP2, DHCR24, SORBS3, KLHL5, NOSTRIN, SPRY2, TRIP11, CDC42SE2 |
| GO:0005925 | focal adhesion | 0.00023 | CD81, ITGB4, RPLP1, RPLP0, FAM107A, DIXDC1, RPL10A, TRIOBP, ICAM1, RPL7, RPL7A, RPS17, ALCAM, RPS19, RAC2, ITGB8, TGM2, KLF11, PDCD6IP, RPS5, ITGA2, ADAM10, RPS3A, ADAM17, ITGA8, NUMB, ARHGEF2, PLEC, AHNAK, RPL12, PXN, ASAP3, TWF1, HSP90B1, DPP4, PDLIM1, IL1RL1, CNN1, FLRT2, GNA12, PACSIN2, CD59, RPS2, CD99, CLASP2, HSPA8, HSPA5, SPRY4, SORBS3, ARHGAP31, DLC1, GNB2, TADA1, CSPG4 |
| GO:0070062 | extracellular exosome | 0.00031 | APP, ICAM1, SMC2, LGALS3, ALCAM, PNP, RPS19, FTH1, TNFSF10, DIP2B, VPS36, GOLGA7, TBC1D10A, CDK5RAP2, GBP6, OMD, DICER1, HDHD2, UPB1, RNASE1, CRISPLD2, RPL24, SERPING1, TAGLN2, CFB, MTMR2, SLC22A5, TMEM63A, STXBP3, PTTG1IP, ACTR3C, HSPH1, FLRT2, RACGAP1, NEU1, SLAMF6, ST3GAL6, ATP6V0A4, PROM1, CP, VASN, HNRNPM, TF, BPIFB2, TMEM98, NAPEPLD, RPS20, SERPINA3, HBS1L, PYGB, ACY3, SLC44A1, SLC44A2, RPLP0, HBB, PYGM, PYGL, JCHAIN, VMO1, MMP24, KRT28, RAC2, TIMP1, CLIC1, RAB8A, CCT2, HBZ, ANXA2, ANXA3, BHMT2, CACYBP, RHOC, SERPINB8, RENBP, APRT, UGDH, CEACAM5, TCP1, ANGPTL2, PMP2, ZNF114, AHNAK, SLC20A2, IDUA, RPL12, CAPG, GNAI1, IGKC, MGAT5, GPC1, CD59, RPS2, RPS27A, CCT5, CCT4, MON2, SLC16A1, GCA, PLEKHA1, RFTN1, UBE2G1, FABP3, ITCH, QPCT, HNRNPA2B1, CFHR3, SLCO4C1, C16ORF89, AGRN, PLCD1, ALDH1L1, ATP8A1, CD81, CSE1L, TM7SF3, RPL10A, COMT, DMBT1, CDH1, CFL2, PEBP4, C1RL, LAMP2, COTL1, ENPP4, PDE8A, TGM2, RPS5, ADAM10, OLFM4, PDIA6, KRT73, ERMN, TOM1L2, S100A6, FSCN1, FKBP4, S100A9, SRSF7, S100A8, COPA, SLC26A2, CNP, RHOBTB3, LTBP3, NID1, ACAT2, HSP90B1, DPP4, MAN2A1, PMVK, PACSIN2, HSPA8, MGAM, HSPA5, HSPA4, ALYREF, DNAJC13, PTPRD, QDPR, DLG1, GNB2, CSPG4, ANXA2P2, SERINC2, ITGB4, SLC2A1, SLC2A5, HEBP2, CA2, ITGB8, HSP90AA1, PDCD6IP, VWF, VPS13C, DCXR, AHSA1, RPS3A, TGFBR3, DNM3, DDX19B, NPC1, TMBIM1, PECAM1, FBP1, PLEC, HSP90AA2P, C1R, RAP1GDS1, LRP2, TTN, DNAJB1, GNA14, MTA1, GNG5, STOM, SLIT2, UGGT1, WASF2, GALNT7, SPAG9, SLC12A2, ERAP1, RPL23A, AKR7L, HSPE1, SOD2, MYO1D, DNAJA1, MYO1B, RAB13, PI15 |
| GO:0032991 | macromolecular complex | 0.00083 | IFITM3, GPSM2, APP, IFITM2, ZFYVE9, HMGB2, DIXDC1, HNRNPU, AHR, NR3C1, CASP9, RASSF2, LBH, DPYSL5, MYC, AKT1, SOX9, VSIG4, CEP97, CEP290, RNF111, SNTA1, ADSL, HSP90AA1, CXADR, ANXA2, ZNF10, OLFM4, CAMKMT, ADAM2, METTL22, RABEP1, ALS2, PARD3, PECAM1, ARHGEF2, FKBP4, BLM, PXK, HDAC2, HSP90AB4P, RBM4B, PSEN1, BCL10, SLC7A1, HIF1A, HSP90B1, STIP1, HSPH1, NCLN, TRA2B, EIF4EBP1, WASF2, UGGT1, UVRAG, SMAD1, MPP4, DTNA, HSPA5, KIDINS220, ZBTB16, PTGES3, RFTN1, USP28, RAD52, ACR, NR4A2, ITCH, ID2, GNB2, UBA3, RADIL, GOPC, PKN1, STT3B, MYD88, RNF220 |
| GO:0043025 | neuronal cell body | 0.00121 | UBXN1, CRTC1, DIXDC1, PPP1R9A, ELK1, BRS3, GHR, ALCAM, DPYSL5, RAB8A, SCN1A, HSP90AA1, KCND1, ANXA3, KREMEN1, COBL, ADAM10, ERMN, ALS2, FEZ1, PARD3, FKBP4, STAU2, TMPRSS3, PSEN1, RTN4, CYGB, FXR1, DNAJB1, GLRA1, DVL1, GNA12, KCNN3, TAC1, SLC38A2, KCNJ2, SLC12A2, PLK3, HOMER1, TGFB3, PLXDC1, SLC8A3, MYO1D, P2RX7, P2RX2, SST, PPP1R1B, STRN, PICALM |
| GO:0072562 | blood microparticle | 0.00134 | SERPINA3, BCHE, HSPA8, HSPA1L, C1S, KDM4D, C1R, ZBTB38, SLC2A1, HBB, CIB2, CP, JCHAIN, TF, DNPEP, IGKC, C1RL, CFHR3, SERPING1, STOM, ANGPTL4, CLIC1, CFB |
| GO:0002199 | zona pellucida receptor complex | 0.00184 | CCT2, HSPA1L, TCP1, ZPBP, CCT4 |
| GO:0005634 | nucleus | 0.00245 | IPO13, RB1, HNRNPU, TESK2, RBPJ, ELK1, SMC2, RPL7, CRHBP, KIAA1109, ZMIZ1, MYC, ZMIZ2, GPBP1, AKT1, DIP2B, VPS36, RNF111, MT1HL1, STARD9, ZNF10, DICER1, EML3, RFX4, RFX5, TXNIP, ZSCAN23, NHLRC1, PRKD1, CLOCK, ZNF397, URI1, MTMR2, SYCP2L, ZNF22, BCL10, AAAS, HIF1A, CYB5R2, HSPH1, AFMID, S100A12, HOXC9, ZNF146, NKX2-2, ZBTB7C, CDK18, PLK3, HACE1, PLK1, RAB3IP, NR1D2, A1CF, ST18, ACR, SETX, FAM220A, HNRNPM, TDP1, NOSTRIN, HBP1, ZNF257, CPEB2, SERPINA3, GMEB1, ZNF131, ZNF251, RPLP0, ZNF44, CTCF, PTH1R, PHF7, TRIOBP, ZNF280D, IFIH1, ING4, TNKS1BP1, TIMP3, NELFB, TEAD3, TIGD2, ZNF362, ANXA2, HSBP1, TET1, EPS15L1, MED29, TEX19, PHF20L1, ITPKC, MED25, MED20, RARB, HCLS1, PLIN2, PMP2, RAPGEF5, ZNF114, ZNF234, PPARD, AHNAK, FAM214B, FOXS1, PLAGL2, PCGF1, RPS2, ZNF468, RPS27A, SF3B1, RUNX1T1, TIGAR, HSPA1L, STAT3, SAMD4B, ELP2, ZNF77, HEYL, FABP3, ZNF219, CENPL, ZNF217, PKN1, CRLF3, PLCD4, YPEL2, ZNF330, ZNF571, ZBTB24, CSE1L, GMNN, ATN1, FAM107A, HIKESHI, AHR, RPL10A, MT1DP, SALL3, PAPOLG, PIM1, MACROD1, PAPOLA, OIP5, HMGN2, MAF1, ZNF202, HMGN1, ZNF442, ZNF441, TLE4, IGFBP3, ZBTB38, HARBI1, USP3, RPS6, THOC1, PRPF4B, MYT1, MOB3B, MSH2, S100A6, SNRNP27, ANAPC5, S100A9, ZNF433, SRSF7, S100A8, ZNF311, SNRPB, BLM, DNMT1, BIRC8, GLIS3, SPATA4, FOXO1, HSP90B1, STIP1, ZNF876P, BAG3, STK36, SMYD2, EIF4EBP1, ZNF787, EVI5, TIA1, ZBTB14, FANCL, ZBTB16, ESRRB, DHCR24, FOXN4, COPS3, LSM7, RGCC, BCL6, CNOT2, ZFP64, TADA1, ZNF536, MDM4, ANXA2P2, MYD88, ZNF772, PIWIL2, CALCOCO2, AKAP8L, PIK3CB, DUSP16, TRO, RPL7A, LBH, DUSP10, ZNF404, ZNF644, UNKL, CEP290, BCAS2, BCAS3, HSP90AA1, EMX2, ITGA2, PATL2, METTL22, ZNF638, HAND1, NUMB, LCOR, DTL, BIRC2, TRIM55, CEBPB, HDAC2, CARNMT1, PPM1H, PBXIP1, PDHB, HDAC9, HDAC7, ZBTB40, DNAJB1, DNAJB6, IGF2BP3, POLR2H, IGF2BP2, MLXIP, SMOX, CABLES1, RPL23A, DNAJA1, RAD52, IMPDH1, NOVA1, ZNF616, KLF9, POLR3G, SMARCAL1, APP, CCNK, TP53RK, CCDC89, TRAF3IP2, CCNH, MT1M, PREP, MT1X, LGALS3, MT2A, MAGEA8, RASSF2, PNP, ZNF607, FTH1, RASSF5, CHEK2, ZNF724, DPF2, SOX9, CCNL2, SKP2, TRIM23, ABT1, AQR, NFKBIL1, KNL1, JMJD8, THAP2, ZPBP, FNDC8, MT1A, RRAGC, MT1G, TRIB3, DNA2, MT1B, AURKAIP1, MT1E, MGST1, ZBTB1, PTTG1IP, FXR1, NUAK1, RACGAP1, DPH3, SCAI, TRMT1L, SRPK2, STAT5A, RABGAP1L, GADD45A, MICAL3, SMARCA5, TRERF1, SRPK1, GADD45G, POLA1, CDAN1, ID2, PPP1R1B, ID4, INTS4, INTS7, ATM, INTS9, HIP1, PYROXD1, ZNF808, CLIC1, TSC22D4, FOXD1, ZHX3, PRMT2, TSC22D3, ZFR, TFE3, CACYBP, RHOC, IGFN1, RAD51B, UGDH, NR5A2, RAD51C, G2E3, ZNF780B, HNRNPH3, DNASE1L1, SATB1, AKAP8, PRKX, CAPG, GNAI1, ARNTL, EXOSC6, ZFP14, HSF1, LMNA, HSF4, METTL4, GPC6, NFYB, PTGES3, MLH1, SORBS3, PER1, RIT2, CAPZA3, STK17A, DLC1, HNRNPA2B1, PRPF31, MPHOSPH8, ZNF354C, OTUD4, NDUFA13, CRTC1, DSCC1, POGK, MAGEB16, NRL, ARID4B, NR3C1, CCND3, CCND1, COTL1, TGM2, ZBP1, OSR1, ADAM10, LIG4, KCTD5, KRT73, CAMKMT, CCNE2, TBL1XR1, CARD19, DNAJB11, NUPR2, PPID, PXK, UBA6, YTHDC2, PSEN1, PDS5A, ACACB, NCKIPSD, DEAF1, SUDS3, RAI2, BCL9L, HSPA8, SMAD1, PDHA1, PNPT1, HSPA5, HSPA4, TGFB3, ALYREF, SLC52A3, AZIN2, CLK4, PHOX2B, CLK1, PAN2, NR4A2, NR4A1, DLG1, UBA3, TCF4, PICALM, USP37, THRA, USP32, NGLY1, HSPB7, HP1BP3, HMGB2, HMGB3, CMAHP, CHD2, CHTF18, YBX3, GHR, RBM3, CASP9, ZC3H7A, PPP2R5E, POLL, RBM6, STPG1, KLF11, TTC37, MKKS, USP48, OLIG3, EED, FIGLA, DCXR, RPS3A, IRAK3, KLF15, ETV5, CCNA2, CCNA1, DDX19B, CREB1, ALS2, OAS1, NCBP3, FBP1, FAN1, SLFN5, RMND5A, RASD1, MTA1, DNPEP, MCRIP2, TRA2B, ERBB2, TRA2A, MKNK2, ATOH8, RGS20, MAPK4, GBP4, TCF7L2, KDM4C, KDM4D, TAF13, SWT1, LIMK2, ZNF181, ZIC5, USP28, U2SURP, RPS6KB1, TEF, SPRY2, NAA15 |
| GO:0005783 | endoplasmic reticulum | 0.00291 | ERO1B, APP, POGLUT1, LPGAT1, ATP8A1, ZDHHC6, MSMO1, IFIT2, SLC39A6, PIEZO1, TGM2, STARD4, RPS6, CNPY1, ACSL5, VASH1, MR1, PDIA6, JMJD8, PDIA4, ERN1, DNAJB11, RPL24, NHLRC1, SLC27A3, PLPP2, INSIG2, INSIG1, YTHDC2, GPR85, MGST1, TMTC2, PSEN1, CYP19A1, RTN4, HSP90B1, KLK6, TMTC4, UGT8, CYB5R3, AGMO, ARSG, PROM1, HHATL, UVRAG, HACE1, HSPA5, EPHX3, DHCR24, ABCA8, A1CF, SRPK1, FA2H, SQLE, CCDC88A, DLG1, TMEM98, RAET1L, STT3B, ULBP2, UBXN1, RNF13, GRIK5, RPLP0, PCSK6, USE1, PIK3C2B, HYAL2, ADAMTS9, EMD, SLC36A1, ELOVL1, VWF, AHSA1, RPS3A, ELOVL6, DUS2, LRTOMT, NPC1, OAS1, ASPH, TMBIM1, ITGA8, TRIM59, PLIN2, PLIN1, MAN1B1, DNASE1L1, STAU2, TMPRSS3, RAP1GDS1, ATL2, MOSPD2, ATP10B, ATP10A, HMGCR, LRP2, GLRA1, EXTL2, ERMP1, STOM, METTL7A, UGGT1, ERAP1, FMO5, FIG4, RNF148, SCD, MGAT4B, PLP2, PLCD4 |
| GO:0005886 | plasma membrane | 0.00302 | CNTFR, MRGPRF, OR4Q3, GRPR, GLDN, ALCAM, GPBP1, AKT1, DIP2A, CXADR, OR11G2, MCEMP1, SYTL4, FEZ1, PARD3, PRKCQ, PRKD1, CFB, ZNF397, CDC42SE1, NLRX1, FPR1, CFP, ADH7, SLC5A3, UGT8, PPP4C, FLRT2, S100A12, PROM1, RHBDL2, MCTS1, ST14, HOMER1, SLC16A11, ABCA8, CP, TMEM98, TDP1, GPR183, NOSTRIN, PLCH1, CDC42SE2, PIGN, KCNC3, GPR21, PTH1R, HIF3A, PHF7, TNKS1BP1, KCND1, ANXA2, ANXA3, NIPA1, ELMOD3, SLC39A10, EPS15L1, SLCO4A1, HCLS1, PLIN2, RAPGEF5, EXOC2, SLC29A2, EXOC1, PLPPR1, AHNAK, SLC7A1, SLC7A2, RHD, GLRA1, MICALL2, RPS27A, JAM3, P2RY12, BCHE, SLC16A1, HSPA1L, OR10G4, STAT3, PLXDC1, SLC8A3, SLCO4C1, PLP1, GOPC, PLP2, CRLF3, AGRN, PLCD4, PLCD1, DGKG, ATP8A1, TM7SF3, GRB14, CDH1, LAMP2, PIM1, SLC39A6, PLS3, SLC16A7, MAF1, SLC39A1, SLC16A3, KCNH2, MYLIP, LHCGR, SEMA6D, HARBI1, GPR75, OLFM4, SLC9B1, ADAM2, FRMD6, MORF4L2, SLCO2A1, S100A6, OR9A2, ADAM8, CLEC4E, S100A9, S100A8, SHC4, SHC2, KCNE1, SLC26A2, CNP, GPR85, CUL1, RHOBTB3, TNFRSF11B, HSP90B1, BAG3, SLC38A2, MPDZ, CLCA4, DRD4, SLC14A1, CD163, HTR1F, OSBPL3, ZBTB16, PTPRD, CNOT2, SLCO1A2, CSPG4, ANXA2P2, MYD88, ITGB4, PIK3CB, ECE2, PIK3C2B, SGCE, PSTPIP2, ITGB8, SPG11, SLC36A1, HSP90AA1, TRPC3, OVCH1, ITGA2, LANCL1, COBL, TNFRSF1A, LRTOMT, ASPH, NRG4, TMBIM1, IL1RAPL1, PECAM1, ITGA8, NUMB, MTFR1, MET, NLGN3, SLC24A2, CATSPER1, NLGN1, SLC5A11, NTN4, ATP10B, ATP10A, LRP2, TTN, ZNRF3, FCHO1, SLITRK1, LRRC8C, LRRC8D, LRRC8A, KCNJ2, SLC12A2, KCNJ10, SEMA4D, ERAP1, KCNJ15, KCNJ16, CPPED1, MYO1D, P2RX7, MYO1B, TSPAN14, P2RX2, KLF9, RAB13, IFITM3, APP, OR9K2, IFITM2, TRAF3IP3, SPARC, GBA2, ICAM1, LGALS3, RASSF2, ZNF607, ARL6IP5, PIEZO2, OXER1, PIEZO1, SLC12A6, TBC1D10A, SCN1A, TRIM23, IL1R1, SLC11A1, ARRDC3, COG3, OMG, ACSL5, CD109, ANTXRL, TRIB3, OR6C4, PLPP2, EPHA3, GYPA, SLC22A5, TMEM63A, STXBP3, SDC2, MGST1, LPAR2, NKAIN2, NEU1, TSPAN5, SLAMF6, ATP6V0A4, KCNN3, DTNA, SLC10A2, MICAL3, IFNLR1, VASN, SRPK1, CCDC88A, ATP4A, TF, CDAN1, LPAR6, RAET1L, OR6A2, RAB40AL, SLC44A5, C2CD5, SLC44A3, SLC44A1, SLC44A2, GRIK5, GRIK1, VLDLR, PCSK6, BRS3, GLIPR1, HYAL2, CLDN24, RAC2, OR5AP2, CLIC1, RAB8A, TNFRSF12A, KREMEN1, RHOC, ANO4, OSMR, IL17RC, NCR1, ADRB3, CEACAM5, TLCD1, OR4C3, OR4A15, SLC43A2, SLC20A2, ADH1B, NOXO1, STX11, GNAI1, C10ORF90, RELN, IGKC, CXCR1, GPC1, CXCR2, CD59, GPC6, MPZL2, MPP4, GCA, MOG, SLC31A2, PLEKHA1, GCGR, RFTN1, LILRB3, ITCH, RIT2, STK17A, MPHOSPH8, PNPLA2, F2RL3, ITPRIP, OR5K3, CRTC1, CD81, OR5K1, MSMO1, COMT, RGS5, IL18RAP, ENPP2, COTL1, ENPP4, IL6R, CA14, TGM2, IL4R, CD93, SLC30A4, ADAM10, MR1, PDIA6, KCTD7, ADAM17, ELMO1, CERCAM, ABCG2, SIDT2, OR1A2, RASL12, PXK, PXN, DAPP1, PSEN1, CACNA1C, PLD1, NID1, PDS5A, SLC1A7, RTN4, RASGRP3, PLD2, DPP4, OR2AK2, PACSIN2, TAC1, GAS2L2, CD99, MARK2, OR2B3, GGT5, HSPA8, MGAM, HSPA5, CNTN6, TGFB3, SLC52A3, DNAJC13, DLG1, LRP10, ULBP2, PICALM, GPSM2, MEGF10, SLC2A1, SLC2A5, USE1, GHR, GRM3, MFSD6, SPRED1, CA2, BLNK, ABCC5, DCXR, PLA2G4C, ARAP3, IRAK3, DNM3, CDH12, ZP3, PLEC, HSP90AB4P, MOSPD2, ABHD17C, ASAP2, OR8S1, GNA14, IL1RL1, RASD1, ERBB3, GNG5, SH3TC2, ERBB2, KLRC4-KLRK1, GNA12, RGS20, STOM, PDE6B, EPS8L3, GBP4, CLASP2, KLRG1, PIK3IP1, ABHD17B, SPRY2, ACKR1 |
| GO:0016324 | apical plasma membrane | 0.00364 | CNTFR, KCNE1, SLC26A2, ACY3, SLC22A5, STXBP3, STK39, SLC2A1, LRP2, PSEN1, PTH1R, PLD1, SLC2A5, SLC7A1, DPP4, ERBB3, HYAL2, ERBB2, PRKG2, ATP6V0A4, PROM1, MPDZ, SLC16A3, IL6R, CLCA4, SLC12A2, MGAM, SLC16A1, ABCC5, CYP4F2, SLC10A2, SLC52A3, OSMR, OCEL1, ATP4A, DLG1, ADAM17, TF, P2RX2, CEACAM5, PARD3, GJB6, CSPG4, ABCG2 |
| GO:0101031 | chaperone complex | 0.00502 | HSPA8, STIP1, BAG2, BAG3, PTGES3, HSF1, DNAJB11 |
| GO:0005938 | cell cortex | 0.00783 | GPSM2, C2CD5, ANXA2, CTTNBP2, KCNC3, PXN, PLA2G4C, MICAL3, RHOBTB3, COBL, PSEN1, RHOC, GNAI1, ERMN, MYO1D, ANLN, ITCH, PARD3, FSCN1, RAC2, PRKD1, FRYL, CLASP2 |
| GO:0043197 | dendritic spine | 0.00783 | APP, MTMR2, NLGN1, KCND1, HOMER1, CTTNBP2, ADAM10, ABHD17C, PPP1R9A, GRM3, DNM3, SLC8A3, FXR1, DNAJB1, EEF2K, ABHD17B, ALS2, DVL1, SYNDIG1, DIP2A, STRN, KCNJ2, RAB8A |
| GO:0009986 | cell surface | 0.00787 | APP, SPARC, ITGB4, HILPDA, HNRNPU, CLEC9A, PCSK6, GLDN, ICAM1, GHR, LGALS3, HYAL2, SLC39A6, ITGB8, DIP2A, ADAMTS9, KCNH2, CD93, ANXA2, SEMA6D, ITGA2, ADAM10, IL17RC, PDIA4, TNFRSF1A, TGFBR3, ADAM2, GREM1, ADAM17, ANTXRL, CEACAM5, CD109, IL1RAPL1, ITGA8, ADAM8, MET, NLGN3, KCNE1, NLGN1, SDC2, LPAR2, PSEN1, LYZL6, DPP4, GPC1, KLRC4-KLRK1, CXCR2, CD59, LRRC8A, SLIT2, PROM1, GPC6, P2RY12, HSPA5, TGFB3, NRDC, VASN, DCBLD2, TSPAN14, TF, RPS6KB1, CSPG5, CSPG4, ULBP2, PICALM |
| GO:0098793 | presynapse | 0.00868 | NLGN3, HSPA8, NLGN1, HIP1, KCNJ10, STXBP3, SLC2A1, SLC1A7, BTBD8, PLD2, RAB33B, P2RX7, DNM3, FXR1, NR4A1, KIAA1109, DVL1, NOSTRIN, SLC29A2, SH3GL1 |
| GO:0005902 | microvillus | 0.00883 | BBS2, CNP, DCXR, SLC10A2, MYO1D, MYO1B, CA2, HYAL2, FSCN1, KIF13B, PROM1, TBC1D10A, JAM3 |
| GO:0035579 | specific granule membrane | 0.00885 | TMEM63A, CD93, ATP8A1, SLC44A2, MOSPD2, ADAM10, MCEMP1, SLC2A5, PLD1, TSPAN14, SLCO4C1, TMBIM1, STOM, ADAM8, CD59 |
| GO:0044297 | cell body | 0.00891 | SLC12A2, CCT2, CXADR, KCNJ10, HSPA1L, RPS6, CACYBP, BTBD8, RIT2, GNB2, TCP1, SYNDIG1, CCT5, CCT4 |
| GO:0071682 | endocytic vesicle lumen | 0.00949 | CSF3, HSP90AA1, SPARC, HSPH1, HBB, HSP90B1 |
| GO:0005874 | microtubule | 0.00989 | DYNC1I2, AKNA, CNP, STAU2, PBXIP1, KLC4, CRHBP, TUBA1B, MTA1, HSPH1, KIF19, RACGAP1, BAG2, RASSF5, DVL1, KIF13B, EMD, CCT5, CDK5RAP2, CCT4, CLASP2, APC2, CCT2, TUBE1, MID1, EML2, DNM3, SLC8A3, TTLL7, DLG1, FEZ1, TCP1, MAP6D1, RADIL, ARHGEF2, TUBGCP4, FKBP4, TRIM55 |
| GO:0000139 | Golgi membrane | 0.01008 | ST6GALNAC1, TRAF3IP3, PGAP2, RNF13, GALNT15, SLC2A1, GCC1, ZDHHC8, LDLRAD4, ECE2, LFNG, AP1G1, HYAL2, DENND5A, GOLGA7, SLC35A5, RAB8A, TRIM23, COG7, COG6, ENTPD4, GLCE, COG4, COG3, LMTK3, COPZ2, ADAM10, MR1, ZDHHC17, TNFRSF1A, RAB33B, B3GNT9, ADAM17, RNF125, SLC9A8, B3GNT2, CHST5, CHST3, TMEM87A, COPA, GCNT1, PSEN1, PLD1, MAN2A2, CHSY1, MAN2A1, MGAT5, ST3GAL5, ST3GAL6, CD59, GBP4, ARFGEF1, GALNT7, HACE1, B3GALT4, DHCR24, HS3ST5, FIG4, MPHOSPH9, MGAT4B, UST, NAPEPLD, CSPG5, TRIP11, GOPC |
| GO:0016363 | nuclear matrix | 0.01087 | ARFGEF1, BLM, CEBPB, SPARC, SATB1, AKAP8L, THOC1, ATN1, AKAP8, HNRNPU, SRPK1, POLA1, HNRNPM, CFL2, LMNA, HNRNPA2B1, RUNX1T1 |
| GO:0016328 | lateral plasma membrane | 0.01091 | SLC12A2, GPSM2, SLC16A1, DLG1, GJB1, ERBB3, CDH1, RAB13, GNA12, DVL1, SLC16A3, MARK2, SNTA1 |
| GO:0016323 | basolateral plasma membrane | 0.01122 | STXBP3, CD81, MEGF11, STK39, SLC2A1, PTH1R, SLC7A1, ERBB3, CA2, ERBB2, SLC12A6, SLC16A3, WASF2, SLC12A2, SLC14A1, ST14, SLC16A1, CXADR, KCNJ10, ANXA2, ABCC5, KCNJ16, ABCA8, MYO1D, DLG1, CEACAM5, SLCO4C1, NUMB, SLC29A2 |
| GO:0017119 | Golgi transport complex | 0.01379 | COG7, COG6, COG4, COG3, DNAJC28 |
| GO:0043202 | lysosomal lumen | 0.01393 | HSPA8, CSF3, HSP90AA1, IDUA, SDC2, OMD, LIPA, GALC, GPC1, LAMP2, NEU1, CSPG5, CSPG4, AGRN, GPC6 |
| GO:0009925 | basal plasma membrane | 0.01405 | P2RY12, SLC12A2, TF, SLC16A1, ERBB3, ITGB4, CD81, ERBB2, MET, SLC7A1 |
| GO:0000793 | condensed chromosome | 0.01436 | ESRRB, SMARCA5, HMGB2, AKAP8, LIG4, CTCF, SMC2 |
| GO:0043209 | myelin sheath | 0.01502 | MYO1D, HSP90AA1, CA2, ERBB2, PLP1, PMP2, ERMN, PLEC |
| GO:0031901 | early endosome membrane | 0.01593 | IFITM3, MON2, MTMR2, ZFYVE9, DNAJC13, LDLRAD4, STAM, PSEN1, MR1, FIG4, ITCH, ZFYVE16, RABEP1, CLCN4, SNX27, SNX16, SYNDIG1, NAPEPLD, PLPP2, STAM2, MARCHF3, SNX5, SH3GL1 |
| GO:0030424 | axon | 0.01627 | APP, MTMR2, HEPACAM, STAU2, KCNC3, LRP2, PSEN1, COMT, GLDN, FXR1, GRM3, ALCAM, BAG2, CA2, KIF13B, DIP2B, MAF1, TAC1, SLC38A2, SPG11, SCN1A, HOMER1, ANXA3, CNTN6, LMTK3, COBL, ADAM10, AZIN2, KLHL24, BTBD8, DNM3, SLC8A3, SETX, MYO1D, CREB1, FEZ1, IL1RAPL1, EIF4G2, PLEC |
| GO:0016942 | insulin-like growth factor binding protein complex | 0.01656 | IGFBP5, IGFBP3, IGF1 |
| GO:0001772 | immunological synapse | 0.01689 | LGALS3, DLG1, ALCAM, PDCD6IP, SNX27, CD81, PRKCQ, BCL10, ICAM1 |
| GO:0043231 | intracellular membrane-bounded organelle | 0.01767 | HIP1, SPARC, ATP8A1, RNF13, CALCOCO2, ZFYVE9, ECI2, PIK3CB, LDLRAD4, COMT, OSBPL11, LIPA, PIK3C2B, GRB14, AP1G1, GPBP1, DPF2, RAC2, OIP5, CCNL2, MAF1, ADAMTS9, PDCD6IP, COG7, ADAM10, RHOC, KLF15, ZDHHC17, RAD51C, RNF125, ZFYVE16, RABEP1, RRAGC, ZNF638, TMBIM1, G2E3, FAR1, AURKAIP1, STAM2, CLOCK, MTMR2, AKNA, TMEM63A, SAMD9, PIK3R4, RHOBTB3, ASAP3, STK39, ARNTL, GLRA1, APOL4, UGT8, CYP2B6, MTA1, NEU1, TRA2A, SYNDIG1, PACSIN2, MARCHF3, SNX5, KLRG1, RANBP2, SLC16A1, HSPA5, TGFB3, FANCL, SMOX, OSBPL3, CYP4F2, SMARCA5, EPHX3, DNAJC13, STAM, ABCA8, CYP4F11, FMO5, BYSL, FIG4, SQLE, ITCH, SNX16, NOVA1, TDP1, ATM, PLCH1, NAA15, PICALM |
| GO:0005789 | endoplasmic reticulum membrane | 0.01815 | ERO1B, LPGAT1, PGAP2, ZDHHC6, MSMO1, CDC14C, PAPPA-AS1, FADS3, ARL6IP5, PIEZO1, SLC39A1, ALG8, ACSL5, ALG13, MR1, PDIA6, ERN1, CARD19, PLPP2, COPA, INSIG2, INSIG1, MGST1, TMTC2, PSEN1, PLD1, CYP19A1, RTN4, HSP90B1, PLD2, HSD11B1, CYB5R2, CYP2B6, CYB5R3, FLRT2, AGMO, HHATL, PNPT1, HSPA5, CYP4F2, OSBPL3, EPHX1, SLC16A11, DHCR24, CYP4F11, FA2H, SQLE, DLG1, GJB1, TMEM98, ACER3, CSPG5, ATG2B, RNF13, PIGN, USE1, SPTLC2, RAC2, ELOVL1, PLA2G4C, COPZ2, ELOVL6, RHOC, ASPH, TRIM59, MAN1B1, PIGF, TMPRSS3, ATL2, MOSPD2, ATP10B, ATP10A, HMGCR, USP19, LMF1, EXTL2, ERMP1, NCLN, ZNRF4, LRRC8C, LRRC8D, SPTSSB, CD59, CEPT1, RPS27A, ERAP1, MBOAT1, FMO5, SLC8A3, CLCN4, DHRS9, SCD, JKAMP, PNPLA3, PLP2, PLCD4, PNPLA2 |
| GO:0005765 | lysosomal membrane | 0.01913 | IFITM3, IFITM2, TRAF3IP3, TMEM63A, RNF13, AHNAK, SLC44A2, WDR41, LRP2, VLDLR, PSEN1, PLD1, GNAI1, DPP4, AP1G1, LAMP2, NEU1, SLC38A9, ATP6V0A4, FNIP1, SPG11, TRIM23, SPAG9, SLC36A1, HSPA8, MIOS, ANXA2, ENTPD4, SLC30A4, HPS4, DNAJC13, CP, VASN, CLCN4, NPC1, GNB2, VPS41, TMBIM1, GOPC, SIDT2 |
| GO:0022627 | cytosolic small ribosomal subunit | 0.01921 | RPS17, RPS19, RPS5, RPS6, B3GALT4, RPS20, RPS3A, RPS2, RPS27A |
| GO:0030425 | dendrite | 0.02171 | MTMR2, URI1, NLGN1, UBXN1, CRTC1, GRIK5, RPLP0, LRP2, CACNA1C, COMT, PPP1R9A, ELK1, SGCE, RBM3, CRHBP, GLRA1, RELN, ALCAM, BAG2, DPYSL5, DIP2B, MAF1, SLC38A2, MPDZ, SPG11, DRD4, MARK2, PLK3, HSPA8, KCND1, HOMER1, HTR1F, ANXA3, RPS6, LMTK3, COBL, ADAM10, AZIN2, DICER1, PLXDC1, SLC8A3, TTLL7, ALS2, FEZ1, IL1RAPL1, STRN, GOPC, PLEC |
| GO:0042470 | melanosome | 0.02450 | HSPA8, HSP90AA1, PDCD6IP, HSPA5, ANXA2, CNP, SLC2A1, HPS4, CAPG, PDIA6, PDIA4, HSP90B1, STOM, ANXA2P2, CCT4 |
| GO:0031902 | late endosome membrane | 0.02465 | IFITM3, IFITM2, ANXA2, RNF13, SLC11A1, SLC30A4, ATP10B, OSBPL11, PLD1, MR1, FIG4, CLCN4, NPC1, SNX16, VPS41, LAMP2, SLC38A9, VPS36 |
| GO:0014069 | postsynaptic density | 0.02649 | MTMR2, NLGN1, CRTC1, RPL12, RPLP0, CACNA1C, PPP1R9A, RTN4, RPL7, FXR1, GRM3, DNAJB1, EEF2K, RPS19, DVL1, SYNDIG1, MPDZ, RAB8A, KCND1, HOMER1, SEMA4C, ADAM10, SHISA6, DNM3, SLC8A3, ALS2, ITGA8, STRN, GOPC, MYD88 |
| GO:0030139 | endocytic vesicle | 0.02986 | DPP4, NLGN3, TF, RABEP1, HYAL2, RAB13, LPAR2, PLD1, STAM2, MTSS1, PIK3C2B |
| GO:0042567 | insulin-like growth factor ternary complex | 0.03144 | IGFBP5, IGFBP3, IGF1 |
| GO:0005923 | bicellular tight junction | 0.03224 | CXADR, PDCD6IP, FRMD4B, PLXDC1, YBX3, OCEL1, DLG1, CCDC85C, CCND1, PARD3, CLDN24, RAB13, MICALL2, STRN, ARHGEF2, MPDZ, JAM3 |
| GO:0022625 | cytosolic large ribosomal subunit | 0.03377 | RPL7A, RPLP1, RPL12, RPLP0, RPL24, RPL35, RPL23A, RPL10A, RPL29, RPL7 |
| GO:0005884 | actin filament | 0.03479 | APC2, COBL, TWF1, ARHGAP6, MYO1B, PSTPIP2, GJB6, COTL1, RAC2, PLS3, HCLS1, GAS2L2, MARK2 |
| GO:1905103 | integral component of lysosomal membrane | 0.03540 | NPC1, ATP10B, LRRC8A, SLC38A9 |
| GO:0048471 | perinuclear region of cytoplasm | 0.03724 | IFITM3, APP, CALCOCO2, ATN1, HMGB2, YBX3, WDR44, AP1G1, CDH1, HYAL2, LAMP2, MAF1, CLIC1, TGM2, CDK5RAP2, KCNH2, APC2, HSP90AA1, ANXA2, ITGA2, RPS6, COBL, DICER1, OLFM4, DNM3, RAD51C, INF2, NPC1, S100A6, NHLRC1, FKBP4, EXOC1, PLEC, MTMR2, HSP90AB4P, CNP, YTHDC2, TWF1, PSEN1, BCL10, PLD1, SLC5A3, HSP90B1, RASGRP3, FXR1, RASD1, DNAJB6, TRA2B, ERBB2, LMNA, HSF1, STOM, GBP4, SNX5, ARFGEF1, SPAG9, HSPA8, HHATL, LIMK2, RAB3IP, AZIN2, TPD52L1, SLC8A3, DNAJA1, COPS3, DLG1, MYO1B, TF, RPS6KB1, PRKRA, GNB2, PICALM |
| GO:0005887 | integral component of plasma membrane | 0.04166 | APP, CD81, GRPR, ICAM1, ALCAM, TNFSF10, SLC39A6, SLC16A7, SLC12A6, SLC16A3, SEMA6B, KCNH2, LHCGR, IL4R, CXADR, MIGA1, IL1R1, SLC11A1, SEMA6D, GPR75, ADAM2, ADAM17, SLCO2A1, ADAM8, PLPP2, EPHA3, SLC26A2, GYPA, LPAR2, CACNA1C, PSEN1, SLC5A3, RASGRP3, FLRT2, SYNDIG1, TSPAN5, ST3GAL5, PROM1, SLC38A2, CLCA4, DRD4, CD99, SLC14A1, ST14, CD163, HTR1F, KLRC3, SLC10A2, SLC52A3, SLC16A11, IFNLR1, PTPRD, ATP4A, LPAR6, GPR183, SLCO1A2, CSPG5, CSPG4, KCNK5, SLC2A1, GRIK1, PTH1R, SLC2A5, BRS3, GRM3, GHR, SGCE, MMP24, TRPC3, ABCC5, LANCL1, SLC39A10, TMC7, TNFRSF1A, TGFBR3, NCR1, NPC1, ADRB3, SLCO4A1, CEACAM5, CEACAM4, PECAM1, MET, SLC29A2, SLC25A13, NLGN3, SLC24A2, NLGN1, PLPPR1, SLC20A2, MOSPD2, ATP10A, SEMA3E, SLC7A1, SLC7A2, RHD, GLRA1, GPR151, ZNRF3, ERBB3, ERBB2, KLRC4-KLRK1, CXCR2, LRRC8C, LRRC8D, STOM, LRRC8A, GABRE, KCNJ2, JAM3, SLC12A2, SLC16A1, KCNJ10, SEMA4D, SLC31A2, SEMA4B, GCGR, SEMA4C, KCNJ15, LILRB3, DCBLD2, SLC8A3, P2RX7, TSPAN14, CLCN4, P2RX2, SLCO4C1, GPR149, F2RL3 |
| GO:0005798 | Golgi-associated vesicle | 0.04355 | ACR, APP, VPS41, ADAM10, MAP6D1 |
| GO:0005788 | endoplasmic reticulum lumen | 0.04485 | APP, COLGALT2, SPON1, POGLUT1, SDC2, CFP, HSP90B1, ADAMTSL4, TSPAN5, TIMP1, ARSG, UGGT1, IGFBP1, BCHE, IGFBP5, HSPA5, IGFBP3, ERAP1, ADAM10, CP, PDIA6, JMJD8, PDIA4, TSPAN14, BPIFB2, ADAM17, TF, IL6, DNAJB11, CERCAM, SERPING1, MATN3, PNPLA2 |
| GO:0005832 | chaperonin-containing T-complex | 0.04598 | CCT2, TCP1, CCT5, CCT4 |
| GO:0034663 | endoplasmic reticulum chaperone complex | 0.04598 | HSPA5, DNAJB11, PDIA6, HSP90B1 |
| GO:0090543 | Flemming body | 0.04698 | PDCD6IP, RACGAP1, USP3, MICAL3, CAPG, EXOC2, EXOC1 |
| GO:0016235 | aggresome | 0.04698 | ZBTB14, BAG3, HSPB7, PRKCQ, PSEN1, HOXC9, ZDHHC17 |
| GO:0042383 | sarcolemma | 0.04945 | POPDC3, ANXA2, AHNAK, SLC2A1, CIB2, PSEN1, SLC2A5, SGCE, SLC8A3, DLG1, SGCA, SLC38A2, SNTA1, PLEC |
| GO:0070821 | tertiary granule membrane | 0.04979 | TSPAN14, MGAM, TMEM63A, CD93, SLC11A1, ADAM10, STOM, ADAM8, CD59, MCEMP1, PLD1 |

| GO ID | Term | P-value | Genes |
| --- | --- | --- | --- |
| MF |  |  |  |
| GO:0005515 | protein binding | 0.00000 | TDRKH, C5ORF24, PGAP2, ZFYVE9, TEX43, TESK2, RBPJ, PTPDC1, CRHBP, KIAA1109, DPYSL5, GPBP1, AKT1, SAMD12, STARD4, CXADR, ZNF10, MCEMP1, DICER1, HDHD2, RNASE1, EML2, EML3, ZFYVE16, MTHFD2, MYL2, FEZ1, RFX4, DDIT4, RFX5, TXNIP, FAR1, CLOCK, URI1, MTMR2, NLRX1, MRPL18, FPR1, AAAS, ADAMTS10, CYB5R2, SULT1A1, PCNX4, HSPH1, PRPSAP2, CYB5R3, AGMO, AFMID, SYNDIG1, MCTS1, HACE1, CYP4F2, SETX, FAM220A, PPFIBP2, TMEM98, TDP1, NOSTRIN, STRN, RNF220, ZNF251, RPLP1, RPLP0, CTCF, PTH1R, OSBPL11, PEAR1, PCMTD2, TUBA1B, NHSL2, MMP28, NELFB, PCMTD1, APC2, ELOVL1, NSUN4, NIPA1, ELOVL6, MRPL48, EPS15L1, MED29, DUSP6, TEX19, CDC20B, PHF20L1, MED25, AGPS, MED20, PMP2, PIGF, PPARD, SAMD9, STAU2, NIP7, ATL2, SLC7A1, STYXL1, GLRA1, EXTL2, SNX27, MFN1, MICALL2, PLAGL2, CEPT1, RPS27A, JAM3, UTP6, SLC16A1, TRAPPC10, OR10G4, STAT3, SAMD4B, ZNF77, PLXDC1, DCBLD2, BYSL, HEYL, FABP3, POM121L4P, ZNF219, CENPL, ZNF217, GOPC, PKN1, CRLF3, AGRN, FRYL, YPEL2, ERO1B, ZBTB24, FAM13C, CSE1L, GMNN, LTN1, ATN1, HIKESHI, FADS3, SLC25A48, SMCHD1, PIM1, SLC16A7, ZNF687, NUDT14, SLC16A3, ZNF202, ZNF441, KCNH2, MYLIP, ZBTB38, FBXO17, PRPF4B, OLFM4, FRMD5, FRMD6, MSH2, LIX1, SLC9A8, C18ORF54, S100A6, FSCN1, SRSF3, SRSF6, S100A9, SRSF7, ZNF433, S100A8, SHC4, INO80C, BLM, SHC2, KCNE1, UQCRB, STK39, GPR85, GLIS3, TNFRSF11B, SPATA4, ACAT2, FOXO1, HSP90B1, SOCS3, STK36, PMVK, SMYD2, EIF4EBP1, EIF4EBP2, SLC38A9, SLC38A2, EVI5, HHATL, TIA1, ZBTB14, DEFA6, ZBTB16, NRDC, HPS4, FOXN4, FAM90A1, PTPRD, RGCC, MDM4, TUBGCP4, CLPTM1L, TMEM19, MYD88, KCNK5, PIWIL2, SH2D4A, ITGB4, AKAP8L, PIK3CB, ZNF404, CEP290, SPG11, UNKL, BCAS2, SLC36A1, BCAS3, HSP90AA1, EMX2, ITGA2, DHPS, AHSA1, TNFRSF1A, NPC1, NRG4, TMBIM1, IL1RAPL1, HAND1, LCOR, TRIM59, PPP1R12B, MET, DTL, PPP1R12C, BIRC2, TRIM55, NDUFB9, C1S, C1R, PPM1H, SLC5A11, NTN4, ATP10B, ATP10A, PBXIP1, LRP2, ZBTB40, ZNRF3, NCLN, ZNRF4, FCHO1, SLITRK1, LRRC8C, LRRC8D, IGF2BP3, LRRC8A, IGF2BP2, SLC25A20, KCNJ2, SLC12A2, FEM1A, MMAA, ERAP1, LYSMD1, CABLES1, RPL23A, TPD52L1, MYO1B, TSPAN14, SST, NOVA1, SMARCAL1, SPON1, CCNK, CCDC89, CCNH, ECI2, MT1M, THUMPD2, PREP, ZDHHC6, MT1X, GCC1, ICAM1, LGALS3, MT2A, RASSF2, RASSF4, RASSF5, CHORDC1, DHX58, TNFSF10, OXER1, PIEZO1, ATXN7L1, CCNL2, ATP6V1E2, TBC1D22B, TRIM23, ALG8, PHYKPL, BCS1L, FNDC8, DHX40, MT1A, MT1G, TAGLN2, TRIB3, FLYWCH1, ZG16, MT1B, TRIM11, EPHA3, MT1E, TBC1D9B, TMEM63A, SDC2, PIK3R4, ZBTB1, LPAR2, PTTG1IP, C7ORF50, SEC14L1, RACGAP1, SEC14L4, NEU1, TSPAN5, SLAMF6, SNX5, BBS2, STAT5A, SLC10A2, SMARCA5, OSGIN1, IGF1, POLA1, TF, LPAR6, ID2, PPP1R1B, ID4, INTS4, INTS7, NOL11, INTS9, FBXL4, ACY3, CCDC120, USPL1, PYROXD1, CCDC125, PAK1IP1, PCSK6, HYAL2, NMRK1, RAC2, NXPE3, CCT2, PBRM1, ZHX3, TNFRSF12A, FSHB, ZFR, CACYBP, CCDC117, OSMR, APRT, NCR1, DUS2, NR5A2, GAN, G2E3, ARHGEF2, CRELD1, TLCD1, KIZ, DYNC1I2, SLC43A2, SATB1, INPP1, HEATR1, MRPS31, NOXO1, CAPG, PRKX, STX11, GNAI1, MNT, ZFP14, GPC1, HSF1, CD59, MARCHF3, GPC6, CCT5, CCT4, MON2, MAP3K1, IL31, GCA, PLEKHA1, UBE2G1, ACTL8, ALDH4A1, PER1, ITCH, RIT2, KLHL5, CFHR3, HNRNPA2B1, CCDC167, OTUD4, KLHL36, CRTC1, CD81, ZFAND3, KLHL32, MSMO1, CCER1, NR3C1, RGS5, CCND3, CCND1, DMBT1, PEBP4, ENPP4, DENND5A, IL6R, SH3GL1, TGM2, ZBP1, IL4R, CD93, SLX4IP, SLC30A4, LMTK3, ADAM10, MID1, MR1, ERMN, CCNB1IP1, ERN1, TERB2, ADAM17, CCNE2, DNAJB11, PPID, UBA6, YTHDC2, DAPP1, CACNA1C, PSEN1, PDS5A, PLD1, ACACB, ACACA, RTN4, RASGRP3, PLD2, NCKIPSD, TMEM243, CNN1, DEAF1, PACSIN2, ISOC1, CD99, RAI2, TBC1D15, IVNS1ABP, MGAM, PDHA1, SLC52A3, KLHL24, CLK4, CLK1, PAN2, IL6, UVSSA, GNB2, UBA3, TCF4, STT3B, PICALM, MXD4, NGLY1, HP1BP3, HMGB2, HMGB3, PPP1R9A, CHD2, USE1, TTC27, LMNB2, GHR, CA2, LRRFIP2, SNTA1, TTC37, MKKS, OLIG3, EED, FIGLA, ARHGEF17, PLA2G4C, IRAK3, ETV5, CCNA2, GREM1, CCNA1, C12ORF4, CREB1, ALS2, CHDH, ATG4C, NCBP3, ZP3, FBP1, PLEC, KRTAP21-2, RAP1GDS1, ASAP3, RBM4B, ASAP2, HMGCR, ABHD17C, CYGB, PDLIM1, IL1RL1, TAF1D, MTA1, DNPEP, GNG5, TRA2B, KLRC4-KLRK1, TAF1A, MKNK2, TRA2A, SLIT2, EPS8L3, ARFGEF1, TAF13, MIIP, HS3ST5, SOD2, U2SURP, CLCN4, TEF, QRICH2, NAA15, FAM98A, VIP, IPO13, RB1, CDA, CNTFR, IPO11, HNRNPU, ELK1, SMC2, RPL7, LCE1B, ALCAM, MYC, WDR90, ZMIZ2, DIP2A, RTTN, C2ORF83, CEP97, VPS36, RNF111, CDK5RAP2, DDX18, WSB1, HOGA1, GTPBP1, SYTL4, RNF125, CLPP, PARD3, RPL24, SNORA74A, SERPING1, ZSCAN23, NHLRC1, PRKCQ, PRKD1, FREM1, CFB, GNE, ZNF397, ANAPC13, FICD, PHYHIPL, BCL10, SLAIN1, CFP, HIF1A, FAM124A, PPP4C, S100A12, PROM1, HOXC9, ZNF146, NKX2-2, CDK18, PLK3, STARD13, PRR32, HOMER1, RAB3IP, PLK1, SLC16A11, NR1D2, A1CF, ACR, SQLE, HNRNPM, FA2H, GJB1, GJB6, HBP1, CDC42SE2, SERPINA3, PYGB, GMEB1, WDR41, PYGM, PYGL, PHF7, ADO, CYB561D1, WDR44, IFIH1, TAF7L, ING4, ADAMTSL4, IMPA2, TRIM2, TIMP3, TNKS1BP1, TIMP1, EMD, TEAD3, ANXA2, MIOS, HSBP1, PLLP, ELMOD3, SERPINB8, INF2, SLCO4A1, TCP1, ANGPTL2, HCLS1, PLIN2, PLIN1, ANGPTL4, ZNF114, EXOC2, EXOC1, CCDC90B, WDR27, PLPPR1, AHNAK, RPL12, GCNT1, WDR20, FAM214B, PAPSS2, GPR151, ERMP1, PCGF1, RPS2, METTL7A, PAIP2, SF3B1, DTD2, RUNX1T1, BCHE, TIGAR, HSPA1L, FIG4, SMOC1, PLP1, PLP2, PLCD4, SCRG1, PLCD1, ZNF330, ANKRD37, ATP8A1, FAM107A, AHR, RPL10A, MT1DP, DEPTOR, GRB14, FCGRT, CDH1, CFL2, PHYHD1, LAMP2, MACROD1, PAPOLA, OIP5, MAF1, MB21D2, HMGN2, SLC39A1, SEMA6B, TLE4, IGFBP1, MAP2K3, IGFBP5, RPS5, ANKRD44, IGFBP3, RPS6, THOC1, VASH2, VASH1, THOC2, MOB3B, TOM1L2, MORF4L2, SNRNP27, ADAM8, FKBP4, CLEC4E, SNRPB, DNMT1, CNP, CUL5, INSIG2, INSIG1, CUL1, RHOBTB3, STIP1, KLC4, BAG2, BAG3, DVL1, MPDZ, DRD4, ANKRD28, UVRAG, CD163, FANCL, CCDC14, OSBPL3, DHCR24, CYP4F11, COPS3, LSM7, BCL6, CNOT2, ZFP64, CSPG5, XAF1, EIF4G2, ZNF772, RNF13, CALCOCO2, AMD1, C1ORF174, DUSP16, PIK3C2B, TRO, RPL7A, DUSP10, LBH, SGCA, AP1G1, EMILIN3, PDCD6IP, TRPC3, GGPS1, VWF, SECISBP2L, LANCL1, COBL, PATL2, METTL22, ASPH, NUMB, PECAM1, POLR1E, MTFR1, SFT2D1, CATSPER1, NLGN3, HDAC2, CEBPB, LINC00242, MTFP1, CEBPD, PRR5L, SEMA3E, PDHB, HDAC9, NSL1, HDAC7, TTN, DCAF15, DNAJB1, DNAJB6, DNAJB5, TNNI3, FNIP1, UGGT1, WASF2, SPAG9, KCNJ10, SEMA4D, NCKAP5L, SEMA4C, KCNJ15, P2RX7, DNAJA1, RAD52, EVI2A, VPS41, MGAT4B, RAB13, POLR3G, PPIL3, METAP1, TRMT61B, GLTP, IFITM3, APP, MTRR, TRAF3IP3, SPARC, TP53RK, TRAF3IP2, DIXDC1, PPP2R2A, CSN3, IFIT1, IFIT2, MAGEA8, PNP, RPS19, ZNF607, FTH1, CHEK2, ARL6IP5, PDK4, DPF2, SOX9, VSIG4, SKP2, SLC35A5, GOLGA7, TBC1D10A, ABT1, AQR, COG7, COG6, IL1R1, NFKBIL1, COG4, CMC2, ARRDC3, COG3, ACSL5, YOD1, KNL1, JMJD8, RRAGC, GSAP, DNA2, AURKAIP1, PLPP2, NECAB1, GYPA, RWDD2B, SMIM5, SLC22A5, STXBP3, MAOA, MGST1, TMTC2, TWF1, KLK6, FXR1, NUAK1, DPH3, ATP6V0A4, SCAI, TRMT1L, SRPK2, DTNA, GADD45A, IFNLR1, STAM, TRERF1, VASN, SRPK1, GADD45G, CCDC88A, CDAN1, ZZZ3, AKR1B10, RAET1L, PDE3A, RPS20, ATM, SLC44A5, HBS1L, GFM1, SLC44A3, HIP1, UBXN1, C2CD3, FASTKD1, GFM2, HILPDA, HBB, VLDLR, TRAM2, GLIPR1, KRT28, KIF13B, CLIC1, RAB8A, HBZ, TSC22D4, EDN1, PRMT2, TSC22D3, TFE3, KREMEN1, NME3, RHOC, IL17RC, ZDHHC17, IGFN1, RENBP, MTSS1, RAB33B, RAD51B, RAD51C, BBS10, ADRB3, POC5, CEACAM5, B3GNT2, HNRNPH3, MATN3, DNASE1L1, PRIM1, AKAP8, C10ORF88, NPL, ARNTL, CST1, CXCR1, CXCR2, LMNA, UBTD1, SPTSSB, MAP4K5, CBR4, MPZL2, RANBP2, UBL4B, MPP4, SLC31A2, MTX2, NFYB, IFNB1, PTGES3, MLH1, LILRB3, SORBS3, PRPF39, MPHOSPH9, PRKRA, CR1L, DLC1, QPCT, TRIP11, PRPF31, MPHOSPH8, NDUFA13, ITPRIP, GALNT15, POGK, DSCC1, CIB2, NRL, ARID4B, LZIC, COMT, IL18RAP, COTL1, UNC5CL, MIGA1, LIG4, KCTD5, PDIA6, KRT73, SHISA6, KCTD7, PDIA4, TBL1XR1, ELMO1, CERCAM, STAM2, ABCG2, RASL12, COPA, AKNA, CTTNBP2, PXN, LTBP3, DNAJC28, DPP4, DNAJC21, LONRF3, SUDS3, GAS2L2, MARK2, HSPA8, SMAD1, STIL, PNPT1, HSPA5, TGFB3, HSPA4, ALYREF, EPHX1, AZIN2, DNAJC13, TDRD7, DNAJC15, NR4A2, NR4A1, DLG1, RADIL, ULBP2, LRP10, GPSM2, ACADVL, USP37, COLGALT2, THRA, MEGF11, USP32, TSEN15, HSPB7, SLC2A1, SLC2A5, CHTF18, YBX3, CKS1B, CASP9, RBM3, HEBP2, MFSD6, SPRED1, PPP2R5E, BLNK, POLL, RBM6, SRGN, KLF11, USP48, DCXR, IQCE, ARAP3, RPS3A, KLF15, TGFBR3, DNM3, DDX19B, RABEP1, OAS1, FAN1, MOSPD2, THBS2, USP19, GNA14, RASD1, RMND5A, ERICH1, ERBB3, MCRIP2, ERBB2, GNA12, RGS20, STOM, BUB3, GBP4, MAPK4, CLASP2, KLRG1, TCF7L2, KDM4D, CMTM5, LIMK2, SPRY4, PPP2R3A, USP28, HSPE1, DLK1, SH3YL1, RPS6KB1, PIK3IP1, SCD, SPRY2, CMTM2 |
| GO:0051879 | Hsp90 protein binding | 0.00013 | SLC12A2, PTGES3, AHSA1, AHR, NR3C1, HIF1A, USP19, ARNTL, ERN1, STIP1, HSF1, CHORDC1, PPID |
| GO:0019901 | protein kinase binding | 0.00039 | USP37, CCNK, PPP1R9A, NR3C1, CKS1B, GHR, CASP9, CCND3, SPRED1, RPS19, CCND1, CHEK2, KIF13B, BLNK, RAC2, AKT1, SLC12A6, RAB8A, CDK5RAP2, MAP2K3, RPS6, ADAM10, IRAK3, RHOC, CCNA2, TOM1L2, FRMD5, CCNE2, MSH2, HCLS1, TRIB3, PPP1R12B, KIZ, PPP1R12C, EXOC2, SHC4, SHC2, RHOBTB3, STK39, BCL10, HIF1A, TTN, HDAC7, RACGAP1, DVL1, HSF1, TNNI3, MAPK4, SLC12A2, TCF7L2, SMAD1, MAP3K1, PLK1, STAT3, B3GALT4, NBEAL1, ELP2, POLA1, IBTK, DLG1, RGCC, SPRY2, CSPG4 |
| GO:0051087 | chaperone binding | 0.00046 | SLC12A2, HSPA8, APP, HSPA5, FICD, VWF, PTGES3, AHSA1, LRP2, HSPE1, CP, USP19, DNAJA1, DNAJB1, STIP1, DNAJB6, BAG2, BAG3, DNAJB5, FNIP1, BIRC2 |
| GO:0051082 | unfolded protein binding | 0.00055 | HSP90AA2P, CCT2, HSPA8, ERO1B, MKKS, HSP90AA1, HSP90AB4P, HSPA1L, HSPA5, PTGES3, HSPE1, HSP90AA6P, HSP90B1, ERN1, DNAJA1, DNAJB1, DNAJB6, DNAJB5, TCP1, DNAJB11, UGGT1, CCT5, CCT4 |
| GO:0050786 | RAGE receptor binding | 0.00103 | APP, FPR1, HMGB2, S100A12, S100A9, S100A8 |
| GO:0042802 | identical protein binding | 0.00128 | GLTP, RB1, APP, CDA, HNRNPU, RPL7, LCE1B, CCDC91, ALCAM, PNP, RPS19, CDH1, RASSF5, FTH1, CHEK2, TNFSF10, AKT1, OIP5, MLYCD, SKP2, NUDT14, SH3GL1, TRIM23, TGM2, ZBP1, KCNH2, CXADR, PHYKPL, COG4, MCEMP1, KCTD5, MID1, CCNB1IP1, KCTD7, ERN1, CLPP, PARD3, CERCAM, PRKD1, ABCG2, NECAB1, BLM, GYPA, MTMR2, FICD, SDC2, MGST1, BCL10, ACACB, ACACA, DPP4, PRPSAP2, BAG2, DVL1, PACSIN2, DRD4, SMAD1, STIL, CHKA, TGFB3, ZBTB16, RAB3IP, PLK1, TYSND1, SETX, QDPR, NR4A1, PPFIBP2, GJB1, BCL6, NAPEPLD, UBA3, TCF4, ATM, MYD88, GPSM2, ACADVL, ACY3, GMEB1, CCDC125, GRIK5, AMD1, SLC2A1, PYGL, PPP1R9A, LMNB2, CASP9, GHR, IFIH1, ADAMTSL4, EMILIN3, PRKG2, CEP290, ADSL, HSP90AA1, VWF, ANXA2, GGPS1, EED, HSBP1, DCXR, DHPS, RENBP, ZDHHC17, MTSS1, TNFRSF1A, DNM3, UGDH, CREB1, ALS2, CEACAM5, HAND1, ANGPTL4, ZP3, MET, FBP1, ZNF114, BIRC2, TRIM55, SLC25A13, NLGN1, CEBPD, AHNAK, C1S, C1R, ATL2, PPM1H, C10ORF88, NPL, HMGCR, TTN, GLRA1, GPR151, TAF1D, DNPEP, ERBB3, DNAJB6, TRA2B, ERBB2, KLRC4-KLRK1, LMNA, HSF1, MFN1, HSF4, STOM, LRRC8A, SLIT2, GBP4, KCNJ2, SPAG9, BCHE, SLC16A1, SEMA4D, MMAA, STAT3, SOD2, TPD52L1, RAD52, P2RX7, ALDH4A1, RPS6KB1, SNX16, TEF, IMPDH1, PRKRA, P2RX2, HNRNPA2B1, VPS41, PLP1, PRPF31, GOPC, CRLF3 |
| GO:0045296 | cadherin binding | 0.00163 | AHNAK, TWF1, CAPG, PSEN1, RTN4, DNAJB1, RPL7A, MMP24, CDH1, BAG3, PACSIN2, TNKS1BP1, RPS2, PROM1, MB21D2, EMD, CLIC1, WASF2, MARK2, TBC1D10A, SNX5, SH3GL1, HSPA8, HSPA5, AHSA1, RPL23A, EPS15L1, OLFM4, VASN, ANLN, DLG1, MYO1B, FMNL2, NUMB, FSCN1, CDH12, RPL24, TAGLN2, RPL29, EIF4G2, PLEC, PICALM |
| GO:0044183 | protein binding involved in protein folding | 0.00461 | CCT2, HSPA8, DNAJB1, HSP90AA1, DNAJB6, HSPA1L, HSPA5, TCP1, CCT5, CCT4 |
| GO:0046872 | metal ion binding | 0.00471 | SPON1, ZFYVE9, MT1M, TESK2, MT1X, MT2A, ZNF607, RASSF5, CHEK2, DHX58, CHORDC1, TNFSF10, ZNF724, DPF2, AGFG2, RNF111, MT1HL1, TRIM23, LIMCH1, CMC1, ZNF10, YOD1, DICER1, CACNA2D4, HDHD2, SYTL4, THAP2, ZFYVE16, MT1A, ANTXRL, MT1G, ZSCAN23, NHLRC1, PRKCQ, DNA2, FLYWCH1, PRKD1, MT1B, FREM1, PLA1A, GNE, MT1E, ZNF397, ZBTB1, OMA1, ADAMTS10, NUAK1, PPP4C, RACGAP1, ADAMTS19, DPH3, ARSG, TRMT1L, ZBTB7C, FER1L4, ADHFE1, MICAL3, TRERF1, POLA1, FA2H, ATP4A, PDE3A, TRMT13, ZNF257, RNF220, ZNF131, ACY3, GMEB1, ZNF251, HBB, ZNF44, PHF7, ADO, CYB561D1, ZNF280D, LFNG, ING4, ZNF808, ING3, IMPA2, NMRK1, TRIM2, TIMP3, TIMP1, TIMP4, ZNF362, ZHX3, KCND1, NME3, PHF20L1, G2E3, ZNF780B, ZNF114, ZNF234, PDZRN4, INPP1, PRIM1, PRUNE2, ZFP14, RELN, ERMP1, CARNS1, MICALL2, PLAGL2, PCGF1, CEPT1, ZNF468, RPS27A, RNF130, RUNX1T1, RANBP2, ZNF77, TTLL7, SLC8A3, RNF148, ZNF219, ZNF217, ZNF354C, YPEL2, ZNF330, ZNF571, GALNT15, ZBTB24, LTN1, CNDP1, MT1DP, SALL3, PAPOLG, PHYHD1, ENPP2, PAPOLA, OIP5, ENPP4, ZNF687, MAP3K6, PDE8A, ZNF202, NUDT14, ZNF442, TGM2, ZNF441, MYLIP, ZBTB38, HARBI1, IGFBP3, OSR1, LMTK3, ADAM10, LIG4, MID1, MOB3B, CCNB1IP1, ADAM17, CARS2, ZNF433, ZNF311, IDI1, BIRC8, PXN, GLIS3, GMPR, CACNA1C, ACACB, ACACA, PHF21B, MAN2A2, ZNF876P, STK36, MAN2A1, DEAF1, SMYD2, SLC38A9, LONRF3, ZNF787, CLCA4, DRD4, SMAD1, ZBTB14, FANCL, ZBTB16, NRDC, PXDNL, PAN2, BCL6, ZFP64, ZNF536, MDM4, STT3B, ZNF772, PIWIL2, RNF13, CALCOCO2, AKAP8L, NGLY1, ECE2, ZC3H7A, ZNF404, POLL, ZNF644, UNKL, KLF11, GGPS1, OVCH1, ITGA2, ARAP3, KLF15, OAS1, ITGA8, TRIM59, FBP1, BIRC2, FAN1, ASAP3, ASAP2, HDAC9, USP19, ZBTB40, HDAC7, DCAF15, PDLIM1, GNA14, ZNRF3, RMND5A, ZNRF4, CHSY1, GNA12, MKNK2, PDE6B, TNNI3, BCO2, GALNT7, KDM4C, KDM4D, LIMK2, ZNF181, ZIC5, HSPE1, SOD2, CPPED1, DNAJA1, POLR3B, IMPDH1, ZNF616, MGAT4B, KLF9, VPS41, PDE7A, METAP1 |
| GO:0016538 | cyclin-dependent protein serine/threonine kinase regulator activity | 0.00475 | CCNA2, CCNA1, CCND3, CCNK, CCND1, CCNE2, CCNH, CCNL2, CKS1B |
| GO:0003725 | double-stranded RNA binding | 0.00516 | ZBP1, ZFR2, STAU2, RFTN1, ZFR, HNRNPU, DICER1, IFIH1, DUS2, TUBA1B, OAS1, PRKRA, DHX58, SIDT2 |
| GO:0001103 | RNA polymerase II repressing transcription factor binding | 0.00573 | BBS2, TCF7L2, MKKS, HDAC2, MTA1, BBS10, STAT3, RBPJ, PPARD |
| GO:0070402 | NADPH binding | 0.00693 | QDPR, DHFR, DUS2, MTRR, HMGCR, CBR4 |
| GO:0045499 | chemorepellent activity | 0.00699 | SEMA6B, DPP4, FLRT2, SEMA4D, SEMA4B, SEMA6D, SEMA4C, SEMA3E |
| GO:0030215 | semaphorin receptor binding | 0.00827 | SEMA6B, RIT2, SEMA4D, SEMA4B, SEMA6D, SEMA4C, SEMA3E |
| GO:0004857 | enzyme inhibitor activity | 0.00873 | PRPSAP2, SPOCK3, TXNIP, TIMP3, TIMP1, ANGPTL4, PPP1R12B, RENBP, PPP1R12C, TIMP4 |
| GO:0000400 | four-way junction DNA binding | 0.00918 | RAD51B, BLM, RAD51C, MSH2, HMGB2, HMGB3 |
| GO:0071949 | FAD binding | 0.00960 | CYB5R2, SQLE, ERO1B, MTRR, CYB5R3, AGPS, MICAL3, MTHFR, DHCR24 |
| GO:0004879 | RNA polymerase II transcription factor activity, ligand-activated sequence-specific DNA binding | 0.01016 | NR4A2, NR4A1, NR5A2, THRA, STAT3, ESRRB, RARB, NR1D2, AHR, NR3C1, PPARD |
| GO:0050431 | transforming growth factor beta binding | 0.01029 | TGFBR3, TGFB3, HYAL2, CD109, SPINT3, LTBP3, VASN |
| GO:0008013 | beta-catenin binding | 0.01043 | APC2, TCF7L2, CXADR, PXN, LZIC, PSEN1, FOXO1, NR4A2, CDH1, TBL1XR1, DVL1, NUMB, TCF4, SOX9, BCL9L |
| GO:0030165 | PDZ domain binding | 0.01262 | NLGN1, CXADR, SLC22A5, PLEKHA1, KIDINS220, SDC2, GRIK5, PSEN1, SHISA6, TGFBR3, ADAM17, GNG5, RPS6KB1, TBC1D10A, SNTA1 |
| GO:0015220 | choline transmembrane transporter activity | 0.01426 | SLC44A5, SLC44A3, SLC44A1, SLC44A2 |
| GO:0005138 | interleukin-6 receptor binding | 0.01426 | IL6, ADAM17, ERAP1, IL6R |
| GO:0031994 | insulin-like growth factor I binding | 0.01661 | IGFBP1, IGFBP5, ITGB4, IGFBP3, LRP2 |
| GO:0031072 | heat shock protein binding | 0.01746 | HSPA8, METTL22, HDAC2, DNAJB6, HSPA1L, BAG2, HSPA5, BAG3, HSF1, FKBP4, PPID, CAMKMT |
| GO:0008184 | glycogen phosphorylase activity | 0.01841 | PYGB, PYGM, PYGL |
| GO:0070290 | N-acylphosphatidylethanolamine-specific phospholipase D activity | 0.01841 | NAPEPLD, PLD1, PLD2 |
| GO:0051721 | protein phosphatase 2A binding | 0.01977 | RPS6KB1, GNA12, EIF4EBP1, AKT1, PPP2R2A, STRN, HMGCR, FOXO1 |
| GO:0016887 | ATPase activity | 0.02310 | HSP90AA2P, BLM, HSP90AB4P, ATP8A1, YTHDC2, RHOBTB3, C10ORF88, CHD2, HSP90AA6P, CHTF18, HSP90B1, SMC2, IFIH1, SMCHD1, HSPH1, KIF19, DHX58, CARNS1, KIF13B, CCT5, CCT4, CCT2, HSPA8, DDX18, MKKS, AQR, HSP90AA1, HSPA1L, HSPA5, HSPA4, SMARCA5, MLH1, HSPE1, BCS1L, RAB33B, DHX40, DDX19B, BBS10, MSH2, TCP1, MYH8, DNA2 |
| GO:0031267 | small GTPase binding | 0.02369 | IPO13, CDC42SE1, IPO11, CSE1L, RHOBTB3, GCC1, YBX3, TRIOBP, RASGRP3, AP1G1, RASSF5, DVL1, MICALL2, DENND5A, EVI5, RAB8A, RANBP2, RABGAP1L, HACE1, ANXA2, MICAL3, HPS4, SYTL4, PEX5L, INF2, FMNL2, ALS2, TRIP11, PKN1, ARHGEF2, EXOC2, CDC42SE2, PICALM |
| GO:0005524 | ATP binding | 0.02465 | DGKG, SMARCAL1, NDUFA13, ATP8A1, TP53RK, HNRNPU, TESK2, SMC2, SMCHD1, PAPOLG, CHEK2, DHX58, CHORDC1, PDK4, PAPOLA, PIM1, AKT1, MAP3K6, TGM2, MAP2K3, DDX18, AQR, ENTPD4, STARD9, LMTK3, LIG4, ACSL5, PRPF4B, DICER1, BCS1L, ERN1, DHX40, MSH2, TRIB3, PRKCQ, PRKD1, DNA2, FKBP4, SLC27A3, CARS2, EPHA3, GNE, ABCG2, PXK, BLM, NLRX1, SLC22A5, FICD, UBA6, YTHDC2, RHOBTB3, PIK3R4, STK39, TWF1, HSP90AA6P, ACACB, ACACA, HSP90B1, ACTR3C, NUAK1, HSPH1, STK36, PMVK, MARK2, SRPK2, CDK18, PLK3, HSPA8, CHKA, HSPA5, HSPA4, PLK1, SMARCA5, ABCA8, CLK4, SRPK1, SETX, CLK1, TBCK, ATP4A, UBA3, ATM, PIK3CB, PYGL, CHD2, CHTF18, PIK3C2B, IFIH1, KIF19, EEF2K, NMRK1, KIF13B, PRKG2, CCT2, MKKS, HSP90AA1, MARS2, ABCC5, NME3, IRAK3, RENBP, RAB33B, RAD51B, ITPKC, DDX19B, RAD51C, BBS10, OAS1, TCP1, MYH8, MET, HSP90AA2P, SLFN5, HSP90AB4P, ATP10B, ATP10A, PRKX, TTL, PAPSS2, TTN, ERBB3, ERBB2, MKNK2, CARNS1, IARS2, MAP4K5, CCT5, MAPK4, CCT4, MAP3K1, KCNJ10, HSPA1L, LIMK2, UBE2G1, HSPE1, MLH1, MYO1D, TTLL7, P2RX7, DNAJA1, MYO1B, CLCN4, RPS6KB1, STK17A, P2RX2, PKN1 |
| GO:0035091 | phosphatidylinositol binding | 0.02635 | BCAS3, PXK, HIP1, NOXO1, STAM, PLD1, PIK3C2B, PLD2, TOM1L2, CCDC88A, SH3YL1, SNX27, SNX16, PARD3, STAM2, SNX5 |
| GO:0030544 | Hsp70 protein binding | 0.02904 | ERN1, DNAJA1, STIP1, DNAJB1, CREB1, FICD, STAU2, HIKESHI, PPID |
| GO:0003729 | mRNA binding | 0.03397 | PIWIL2, RBM4B, RPL7, FXR1, MYC, TRA2B, RPL35, IGF2BP3, IGF2BP2, RPS2, PAIP2, SF3B1, SRRM3, AQR, ALYREF, RPS5, SAMD4B, TDRD7, THOC2, A1CF, DHFR, HNRNPM, NOVA1, RPL24, NCBP3, SRSF6, EIF4G3, EIF4G2 |
| GO:0042803 | protein homodimerization activity | 0.04028 | CDA, APP, HIP1, CALCOCO2, CIB2, AHR, PTH1R, JCHAIN, GHR, SMCHD1, IMPA2, CHEK2, AKT1, IL6R, KCNH2, HSP90AA1, ZHX3, PDCD6IP, PRMT2, MIGA1, HMGCS1, ZBTB38, SLC11A1, GLCE, ADAM10, IRAK3, CACYBP, HOGA1, UPB1, MID1, ERN1, GREM1, RABEP1, ADRB3, ALS2, MSH2, CEACAM5, HAND1, S100A6, PECAM1, ABCG2, ZNF397, BLM, CEBPB, FICD, CARNMT1, ZBTB1, HSD17B4, RTN4, DPP4, FXR1, HSD11B1, MAN2A1, SYNDIG1, STOM, SLIT2, GBP4, MAPK4, JAM3, MMAA, DEFA6, GCA, GADD45A, ZBTB16, STAT3, HPS4, TPD52L1, HEYL, CCDC88A, PRKRA, C16ORF89 |
| GO:0008270 | zinc ion binding | 0.04044 | ZNF330, CDA, THRA, ZFAND3, MT1M, LTN1, MT1X, CTCF, NR3C1, IFIH1, MT2A, MMP24, ZMIZ1, CA2, TRIM2, ZMIZ2, DHX58, MMP28, CHORDC1, TNFSF10, ENPP2, TIMP1, ADAMTS9, CA14, TRIM23, BHMT2, USP3, LANCL1, ZFR, TET1, MYT1, MID1, MT1A, RNF125, NR5A2, ZNF638, S100A6, MT1G, RARB, ADAM8, TRIM59, ARHGEF2, MT1B, S100A9, SRSF7, S100A8, TRIM11, TRIM55, BIRC2, MT1E, PPARD, BLM, DNMT1, ADH1B, PRIM1, AKAP8, RBM4B, ZNF22, ADH7, DTX4, GLRA1, MTA1, DNPEP, DNAJC21, S100A12, MARCHF3, ZNF146, KDM4C, DTNA, MAP3K1, ZFR2, ERAP1, ESRRB, NR1D2, ST18, ACR, NR4A2, POLA1, NR4A1, ZZZ3, QPCT, ACER3, NAPEPLD, MDM4, XAF1 |
| GO:0070888 | E-box binding | 0.04063 | PER1, FIGLA, MYC, ATOH8, TCF4, AHR, HIF1A, CLOCK, ARNTL |
| GO:0004386 | helicase activity | 0.04887 | DHX40, SETX, SMARCAL1, DDX18, BLM, AQR, DDX19B, SMARCA5, DNA2, DICER1, CHD2 |
