# Supplementary figures and images for: Exploring the association of addiction-related genetic factors with non-suicidal self-injury in adolescents
Source: Front Psychiatry. 2023 Mar 31;14:1126615. doi: 10.3389/fpsyt.2023.1126615 (PMC10102595; doi:10.3389/fpsyt.2023.1126615)

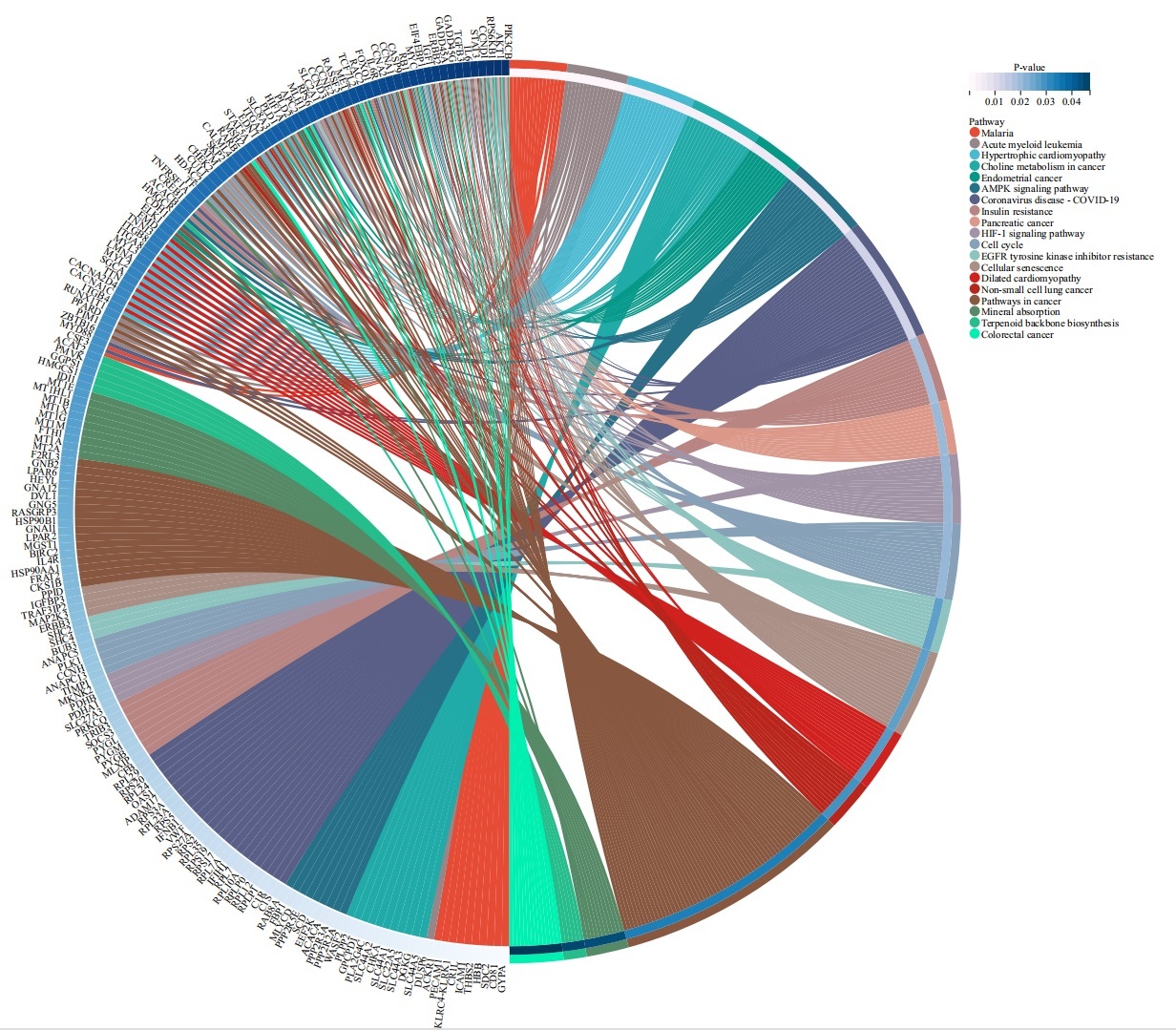

Supplement: Supplementary file 6 [file Image_1.JPEG]
